# Durable multifunctional cotton textiles through conformal ZnO nanocoatings by atomic layer deposition

**DOI:** 10.1039/d6na00078a

**Published:** 2026-09-02

**Authors:** Md Sazid Bin Sadeque, Fatih Bayansal, Mahmoud Aboelkheir, Lindsay Wolverton, Isabelle Reitz, Nishka Avunoori, Rimi Chowdhury, Necmi Biyikli, Tamer Uyar

**Affiliations:** a Fiber Science Program, Department of Human Centered Design, College of Human Ecology, Cornell University 14853 Ithaca NY USA tu46@cornell.edu; b Department of Electrical & Computer Engineering, University of Connecticut 06269 Storrs CT USA; c Institute of Materials Science, University of Connecticut 06269 Storrs CT USA; d Department of Microbiology, College of Arts and Science, Miami University Oxford OH 45056 USA

## Abstract

Atomic layer deposition (ALD) of ZnO enables conformal nanoscale coating on cotton textiles, imparting multifunctional properties for high-performance and smart fabric applications. However, the influence of ALD processing temperature on coating characteristics, wash durability, and functional performance has not been systematically investigated. In this study, ZnO nanocoatings were deposited on cotton fabrics at three different temperatures (room temperature, 50 °C, and 120 °C), and the ZnO growth, textile properties, and multifunctional performances were evaluated accordingly. The ALD-coated cotton exhibited approximately 11-fold improvement in photocatalytic self-cleaning efficiency, achieving almost complete degradation of dye molecules within 15 h, and a 10-fold enhancement in UV-blocking performance, transmitting less than 2.5% of incident UV radiation. The ALD-coated cotton also showed antibacterial activity against both Gram-negative and Gram-positive bacteria while retaining excellent biocompatibility with >96% mammalian cell viability. The ZnO-coated cotton exhibited improved thermoregulatory behavior compared with pristine cotton. Importantly, the multifunctional properties of ZnO-coated cotton were retained after repeated laundering cycles without compromising the intrinsic textile characteristics of the cotton fabric. These results demonstrate the potential of ALD as a low-temperature and wastewater-free sustainable textile finishing technology for the development of durable multifunctional textiles for healthcare, protective, wellness, and smart wearable applications.

## Abstract 2

ALD ZnO nanocoating transforms cotton into a durable multifunctional textile with enhanced self-cleaning, UV-blocking, antibacterial, and thermoregulatory properties while retaining washability and textile performance.

## Graphical abstract

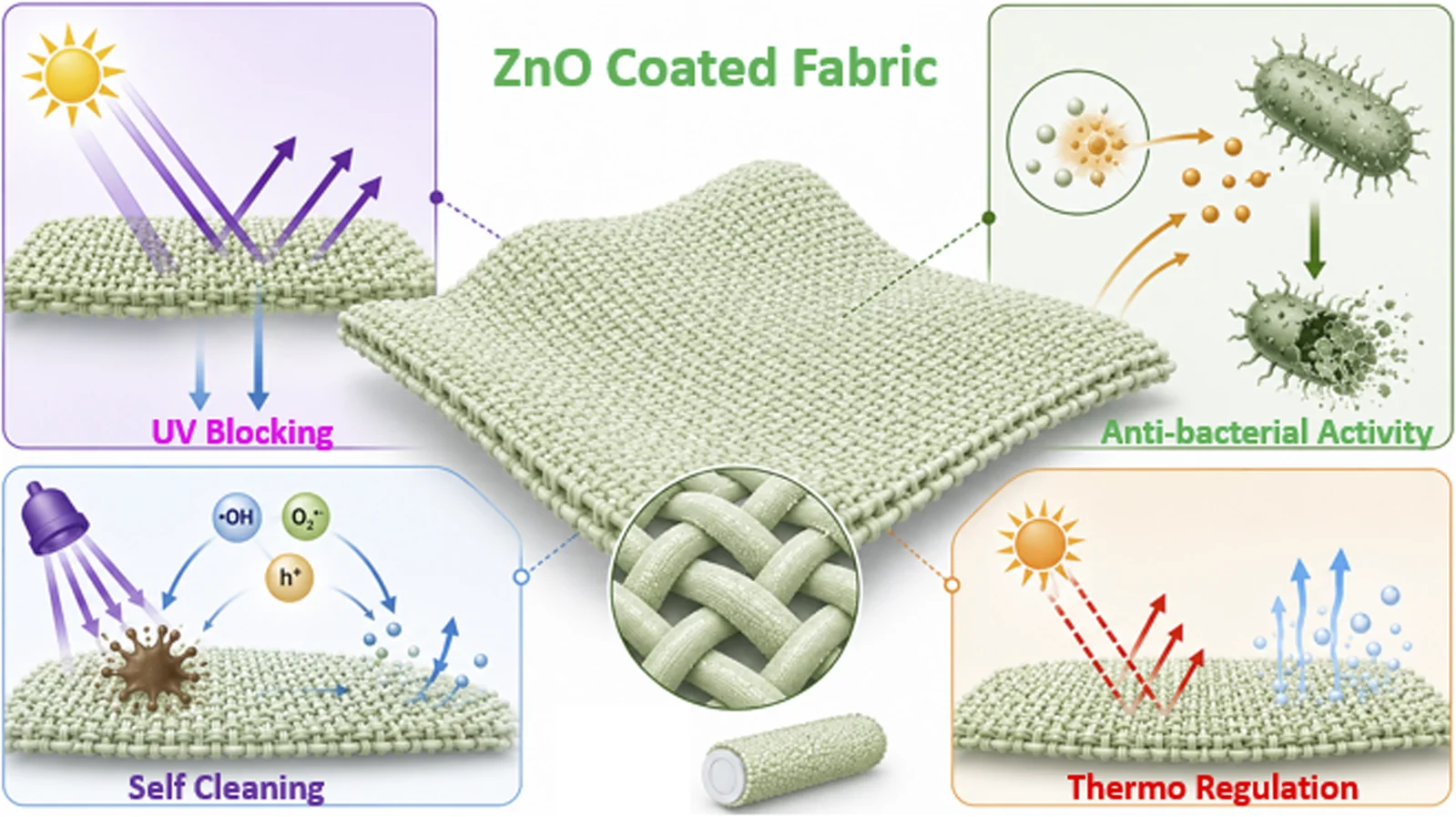

## 1 Introduction

Zinc oxide (ZnO) is one of the most extensively studied semiconducting metal oxides because of its wide band gap of approximately 3.4 eV, high exciton binding energy, high electron mobility, and tunable optical properties.^1^ These characteristics have made ZnO highly attractive for optoelectronic applications such as light-emitting diodes,^2^ UV lasers,^3^ solar cells,^4^ displays,^5^ photodetectors,^6^ and flexible electronics.^7^ In addition to its optoelectronic significance, ZnO has also received growing attention for textile applications because of its multifunctional properties, particularly photocatalytic activity,^8^ antibacterial behavior,^9^ and UV-blocking capability.^10^

A wide range of methods have been used to deposit ZnO on textile substrates. Unlike other textile coating technologies,^11,12^ ALD provides unprecedented atomic-level control of film thickness and uniformity as layer-by-layer coating ensures uniform particle distribution over the entire substrate.^13^ In addition, the surface deposition by the sequential surface saturating reaction of ALD is mostly free of pinholes and mechanically strong on the adherent surface.^14^ Moreover, ALD is less sensitive to fluctuations in precursor concentration and environmental variables, which enables reproducible and scalable deposition performance with batch-to-batch consistent quality control. These features make ALD especially suitable for providing fabrics with multifunctionality while minimally disturbing the intrinsic properties of the textile substrate.

Despite these advantages, most studies on ALD-coated textiles have concentrated on e-textile applications such as sensing, logic, display, memory, and energy-related devices, typically at the level of fibre, yarn, or local fabric patch.^15,16^ Conversely, the multifunctional behaviour of ALD-grown ZnO coatings on bulk textile substrates is comparatively underexplored (Table S1). This difference is likely due to the special requirements of textile products, which need to provide functional performance while maintaining essential textile properties such as softness, flexibility, breathability, drape and aesthetic appeal. Textiles also undergo repeated mechanical deformation, abrasion, and laundering during their service life, and long-term durability is therefore a critical requirement. A key challenge in functional coatings is to develop coating integrity and performance under these conditions while maintaining the inherent properties of the textile. Hence, there is an urgent need for systematic studies addressing not only the functional capabilities of ALD coatings but also their durability, wash resistance, and compatibility with the performance requirements of textiles. Closing this knowledge gap is critical to scale up ALD-based textile technologies from lab-scale demonstrations to commercially viable multifunctional and smart textile products.

Previous studies on ZnO-coated textiles have predominantly focused on single deposition temperatures or single specific applications, such as antibacterial, photocatalytic, or UV-protection textiles (Tables S1 and S2).^17–21^ Consequently, the relationship between ALD deposition temperature and key coating characteristics – including growth behaviour, crystallinity, conformality, multifunctional performance, and laundering durability – has not been comprehensively established. This knowledge gap is significant because deposition temperature is a critical process parameter that governs precursor adsorption, surface reaction kinetics, nucleation density, crystal growth, and film continuity during ALD. Variations in these factors can substantially influence the morphology, coverage uniformity, and functional properties of the resulting ZnO coatings. Moreover, while ZnO offers attractive photocatalytic, UV-blocking, antibacterial, and electronic functionalities, its interaction with textiles is primarily governed by physical adhesion rather than strong covalent bonding. As a result, coating stability under repeated washing and mechanical use remains a major concern for practical textile applications. A systematic understanding of how deposition temperature affects both coating performance and long-term durability is, therefore, essential for optimizing ALD processes and advancing the commercialization of multifunctional ZnO-coated textiles.

In our recent work, we showed that ALD-coated cotton fabric can be used in wearable optoelectronic sensing, indicating the potential of direct metal oxide deposition on conventional textile platforms.^22^ However, the long-term durability of ALD-enabled multifunctional properties under realistic textile usage conditions is not well explored. In an effort to address this important knowledge gap, we have systematically investigated the durability and performance retention of ZnO-coated cotton fabrics deposited by thermal ALD in this work. Thermal ALD was preferred over plasma-assisted deposition because plasma processing can lead to fibre surface roughening, degradation and discolouration, which can negatively impact textile aesthetics and performance. Moreover, a 100 GSM cotton fabric was used for thermal ALD, as its lightweight structure exhibits relatively high native UV transmittance, underscoring the need and opportunity to improve its UV-blocking ability through ZnO coating.^23^ Overall, by linking ALD deposition temperature to coating characteristics, laundering durability, and multifunctional performance, this study establishes key relationships between processing conditions, structural evolution, and the retention of antibacterial activity, photocatalytic self-cleaning, UV-blocking, and thermal-management performance. The effects of ZnO deposition on fabric color, mechanical properties, thermal stability, air permeability, porosity, and water absorbency were also systematically evaluated to assess practical textile compatibility. These findings provide design guidelines for developing durable, washable, and multifunctional smart textiles and advancing ALD technology for next-generation textile finishing.

## 2 Experimental section

### 2.1 Materials and chemicals

Plain weave cotton fabric (100 GSM) and other fabric samples were purchased from Testfabrics, Inc. (West Pittston, PA, USA). Diethylzinc (DEZ, ≥95%) was purchased from Strem Chemicals, Inc. and used as the zinc precursor for the atomic layer deposition (ALD). Rhodamine B (RhB) dye was obtained from Alfa Aesar. Commercial laundry detergent (Persil Deep Clean Liquid, Henkel Corporation, Rocky Hill, CT, USA) was purchased from a local retail store. The SILWICK® wetting liquid (surface tension = 19.1 dynes cm^−1^) was provided by Proous Materials Inc. Rubber balls used in accelerated laundering tests were purchased from Testfabrics, Inc. (West Pittston, PA). The photocatalytic tests used a 100 W UV light source (IP66, Everbeam) with a wavelength of 365 nm. Quartz cuvettes (1 × 1 × 4 cm^3^) were obtained from Cwmiibili. Thermal imaging camera HP96 (HSF Tools) was used to measure the surface temperature. All materials and chemicals were used without further purification or modification.

LB broth and LB agar were purchased from BD Biosciences. Streptomycin sulphate, potassium phosphate, Triton X-100, MTT (3-(4,5-dimethylthiazol-2-yl)-2,5-diphenyl-2*H*-tetrazolium bromide) and dimethyl sulfoxide (DMSO) were from Sigma-Aldrich. Dulbecco's Modified Eagle Medium (DMEM), foetal bovine serum (FBS) and Hanks' Balanced Salt Solution (HBSS) were purchased from Gibco, Thermo Fisher Scientific. All chemicals and reagents used were of analytical grade and used without further purification. The Gram-positive bacterium, *Staphylococcus aureus* (ATCC 25923), and the Gram-negative bacterium, *Escherichia coli* (ATCC 25922), were purchased from the American Type Culture Collection (ATCC) and used for antibacterial assays. The human cervical cancer cell line HeLa (ATCC CCL-2) was also purchased from ATCC for cytotoxicity evaluation.

### 2.2 ALD of ZnO nanocoatings on cotton fabric

ZnO nanocoating was deposited on cotton woven fabric. A fabric swatch of 10 × 10 cm^2^ of 100 GSM bulk cotton fabric was cut out. The sample was introduced into an HCP-ALD reactor (Layerava Plasma Enhanced ALD, Okyay Technologies Inc., Turkey). Before surface deposition, the chamber was evacuated to a base pressure of ∼30 mTorr. The deposition was performed in thermal mode without plasma processing using DEZ (diethylzinc) as the Zn precursor and DI water as the oxidant. The standard recipe used 400 cycles and consisted of a 500 ms DEZ pulse, 30 s N_2_ purge, 500 ms H_2_O pulse, and 30 s purge. The final step of the room temperature process was extended to 40 s for the nitrogen purge to remove any unreacted water vapour. The films were deposited at three different temperatures (room temperature, 50 °C and 120 °C). In the discussion section, these samples are denoted as Cotton (uncoated sample), ZnO@RT (ZnO-coated cotton sample at room temperature), ZnO@50 (ZnO-coated cotton sample at 50 °C) and ZnO@120 (ZnO-coated cotton sample at 120 °C).

### 2.3 Characterization

Surface and morphology of the pristine and ZnO-coated cotton samples were studied using a Zeiss Gemini 500 scanning electron microscope. Samples were mounted on carbon tape and coated with gold–palladium alloy by sputtering. X-ray diffraction (XRD) spectra were collected on a Bruker D8 Advance ECO powder diffractometer with Cu-Kα radiation (40 kV, 25 mA). X-ray photoelectron spectroscopy (XPS) was performed on a Thermo Fisher Scientific Nexsa G2 with a focused Al K monochromatic X-ray source (1486.6 eV). Differential scanning calorimetry (DSC) measurements were performed on a TA Instruments Q2000. Thermogravimetric analysis (TGA) was performed using a TGA analyser (Q500, TA Instruments). Tensile strength of the samples was studied using an Instron universal testing machine. Water absorbance was measured using an M/K GATS. Porosity was determined using a capillary flow porometer CFP 1100 (Porous Material Inc.). The air permeability was determined using a Frazier Precision Instrument air permeability tester (I97A). Colourimetric analysis was performed using X-Rite. Accelerated laundering was performed using Rotawash-SDL Atlas. The absorbance of RhB in the solution with the coated and uncoated samples was measured using a PerkinElmer Lambda 35 UV-vis spectrophotometer. The UV transparency of the samples was measured using a Cary-5000-UV-Vis-NIR-spectrometer.

### 2.4 Laundry test

4 × 2 cm^2^ fabric swatches were cut for the laundry washing test. Each sample was transferred to a separate can with 10 rubber balls. Total liquor volume was prepared for each canister according to the AATCC TM61 1BC test method with a liquid detergent concentration of 0.56%. The test was performed at 31 °C for 20 min. Samples were rinsed and then transferred from the canister into individual beakers. All samples were rinsed three times in DI water for 1 min with occasional stirring and hand squeezing. The samples were then dried in an oven at 50 °C and then stored in a conditioned room for further tests. In the discussion section, washed samples are abbreviated as Cotton-1W/5W (Cotton without coating, 1 wash/5 wash), ZnO@RT-1W/5W (Cotton coated with ZnO at room temperature, 1 wash/5 wash), ZnO@50-1W/5W (Cotton coated with ZnO at 50 °C, 1 wash/5 wash) and ZnO@120-1W/5W (Cotton coated with ZnO at 120 °C, 1 wash/5 wash).

### 2.5 Tensile test

The tensile testing was done under dry conditions in accordance with the 1C-E (cut strip, constant-rate-of-extension) method described in ASTM D5035-11. Three specimens of each sample category were tested. Rectangular specimens of 100 × 20 mm^2^ were prepared from the fabric sample. The specimens were pretreated at room temperature for 6 h and then mounted in a universal tensile testing machine with an initial gauge length of 75 mm and loaded in tension at a constant speed of 300 mm min^−1^ until failure. The applied load and the corresponding extension were continuously recorded during testing. The load-extension data were transformed to stress–strain data using the dimensions of the specimens and the gauge length and stress–strain curves were plotted for each specimen.

### 2.6 Air permeability test

The air permeability test was carried out according to the ASTM D737 Standard Test Method for Air Permeability of Textile Fabrics. The fabrics were cut to a 5 cm diameter, pre-conditioned, and clamped in the test head. The pressure drops across the fabric surface and the corresponding volumetric airflow through the fabric were measured using a manometer and a precision-calibrated nozzle (orifice) system.

### 2.7 Porosimetry test

The pore characteristics of the fabrics were measured using a PMI CFP-1100-AEHXL capillary flow porometer (Porous Materials Inc., USA) in dry-up/wet-up mode. The instrument was used to compare the dry and wet flow curves to determine the pore size distribution and characteristic pore diameters. Circular fabric specimens of 25 mm diameter and 0.22 mm thickness were prepared and loaded into the sample holder. The dry-up measurement began by gradually increasing the gas pressure on the dry specimen and recording the resulting flow rate to generate the dry flow curve. The specimen was then fully impregnated with SILWICK® wetting liquid (surface tension = 19.1 dynes cm^−1^) and retested under the same conditions for the wet flow curve. The measurements were made at a maximum pressure of 40 psi, and a tortuosity factor of 0.713 was used in the analysis.

### 2.8 Gravimetric absorbency testing system

A circular pre-cut fabric sample of 5 cm diameter was pre-conditioned, weighed, and placed on the M/K GATS plate that is connected to a water reservoir. The plate height was automatically adjusted such that zero hydrostatic pressure was created between the fabric and reservoir to allow the water to enter the fabric sample without any external pressure. The test was run for a period of 1000 s.

### 2.9 Martindale abrasion test

The abrasion properties of the coated samples were tested using a Martindale tester (M235) as per test method ASTM D4966-22. A felt layer and a conventional abrasive fabric (140 mm dia) were laid on the testing table and pressed down with a mounting weight to prevent the formation of wrinkles or uneven surfaces. The fabric specimen was cut to a diameter of 30 mm and was mounted in a specimen holder in the face-down position. Since the basis weight of the cotton fabric was less than 500 g m^−2^, a disc of polyurethane foam was placed between the specimen and the metal insert to support the specimen adequately during abrasion. The assembled holder was placed on the Martindale machine, and an appropriate load corresponding to a pressure of approximately 9 kPa was applied, which is suitable for apparel textiles. The machine was then operated for 500 abrasion cycles using a multidirectional rubbing motion.

### 2.10 Colorimetry testing

The colour characteristics of uncoated and ZnO-coated cotton fabrics were measured using an X-Rite spectrophotometer with a D65 illuminant and 10° standard observer (D65/10). Before the measurement, the instrument was calibrated using the white calibration standard supplied by the manufacturer according to the manufacturer's instructions. The uncoated cotton fabric was chosen as the reference standard, and the ZnO-coated samples were analyzed comparatively. The fabric specimens were gently mounted flat on the measurement aperture for complete coverage and to minimise background interference. Reflectance spectra were measured in the visible wavelength range of 360–750 nm and the corresponding CIELAB colour coordinates (L*, a*, and b*), chroma (C*), hue angle (*h*°) and total colour difference (ΔE*) against the reference cotton fabric were computed automatically using the instrument software.

### 2.11 Photocatalytic self-cleaning

The photocatalytic self-cleaning efficiencies of the different ZnO-coated samples were measured under washed and unwashed conditions using a 10 µM RhB solution. The uncoated cotton and ZnO-coated cotton (120 °C, 50 °C, and RT) samples under different wash conditions, no wash, 1 wash (1W), and 5 washes (5W) were cut into 4 × 2 cm^2^ pieces and immersed in a cuvette filled with RhB solution. Next, the samples in the cuvette were irradiated with UV at 365 nm (100 watts). Absorbance spectra of all samples were recorded at 0, 1, 2, 3, 6, 9, 12, and 15 h intervals.

### 2.12 UV blocking

A UV-vis spectrometer was used to measure how much UV light passed through the sample *versus* the incident light. The samples were cut into dimensions of 2 × 2 cm^2^. The instrument was fitted with a DRA-small spot kit, focused on a 4 mm pinhole in transmittance geometry for the measurement.

### 2.13 Qualitative analysis of antibacterial activity

The ZnO-coated fabric samples (unwashed and machine-washed, 1W and 5W) were assayed for antimicrobial activity using a standard disc diffusion method. Fabric specimens (1.5 × 1.5 cm^2^) were prepared and sterilised by UV irradiation for 30 min before testing. Antibacterial activity was evaluated against Gram-positive *Staphylococcus aureus* (ATCC 25923) and Gram-negative *Escherichia coli* (ATCC 25922). Bacterial cultures were grown overnight in Luria–Bertani (LB) broth and incubated at 37 °C with constant shaking at 200 RPM. Fresh LB medium was used to dilute the cultures 1 : 100, and they were grown to an OD600 of 0.5. The bacterial suspensions were then mixed thoroughly and uniformly spread onto LB agar plates (100 mm diameter, >6 mm thickness, and pH ≈ 7.2) with sterile cotton swabs to form a confluent bacterial lawn. The fabric samples were then carefully placed in the middle of the inoculated agar plates. Streptomycin was used as a positive control in this test. 20 µL of a 50 mg mL^−1^ streptomycin solution was placed on a sterile filter paper disc of size 1.5 × 1.5 cm^2^ and placed on a separate inoculated plate. The plates were incubated at 37 °C for 20 h. Subsequently, the plates were analysed and photographed after incubation. A zone of inhibition (ZOI) was defined as the visually clear zone around the fabric sample with well-defined, free-of-bacteria margins. The antibacterial activity of the samples was quantified by measuring the inhibition zone diameter in mm using ImageJ software.

### 2.14 Quantitative analysis of antimicrobial activity

The antibacterial activity of ZnO-coated fabric samples was quantitatively evaluated against Gram-negative *Escherichia coli* (ATCC 25922). Bacteria were grown at 37 °C for 18 h in Luria–Bertani (LB) broth with constant shaking at 200 rpm. The overnight culture was diluted with potassium phosphate buffer to an optical density (OD475) of 0.28, corresponding to approximately 2 × 10^8^ CFU mL^−1^. 5 mL bacterial suspension was transferred to sterile tubes. The suspension was then diluted with potassium phosphate buffer to a final bacterial concentration of approximately 2 × 10 5 CFU mL^−1^. Fabric specimens with a weight of about 50 mg were cut and sterilised by UV irradiation for 30 min before testing. The sterilised fabric samples were then introduced into the bacterial suspensions. For the positive control, 20 µL of streptomycin solution (50 mg mL^−1^) was added to the bacterial suspension. Control tubes without the fabric samples but containing the bacterial inoculum were also prepared and left untreated. All tubes were vortexed thoroughly to ensure uniform contact between bacteria and fabric samples. To evaluate the initial bacterial population to which the fabrics coated with ZnO were exposed, a colony count was performed at 0 h immediately after vortexing. Each suspension was diluted tenfold in serial dilution with a 0.1 mL aliquot and spread in quadruplicate (0.1 mL of diluted suspension) on LB agar plates. The other suspensions were incubated at 37 °C for 1 h under shaking conditions. A contact time of 1 h was chosen to evaluate the rapid antibacterial effect of the ZnO-coated fabrics and to test if a significant reduction of bacteria could be achieved in a short time of exposure, demonstrating the antibacterial effect. However, this short-contact assay was different from long-contact methods, *e.g.*, AATCC 100, which usually measure the antibacterial performance in a period of 18–24 h. After incubation, 0.1 mL aliquots were diluted 10-fold and plated in quadruplicate on LB agar plates. The agar plates were then incubated at 37 °C for 24 h. Following incubation, bacterial colony-forming units (CFUs) were counted. The untreated control after 1 h of incubation exhibited 100% bacterial survival, and the survival of the treated samples was normalised to this control. The antibacterial activity of the ZnO-coated fabric samples was evaluated by measuring the reduction of *E. coli* survivability after exposure to the coated fabric.

### 2.15 Qualitative and quantitative assessment of cytotoxicity

The potential cytotoxicity of the ZnO-coated fabric samples and their effects on cell viability were evaluated qualitatively by phase contrast microscopy and quantitatively by indirect 3-(4,5-dimethylthiazol-2-yl)-2,5-diphenyl-2*H*-tetrazolium bromide (MTT) assay according to the standard protocol of ISO 10993-5 for *in vitro* cytotoxicity assessment. HeLa cells were chosen in this study because of their high sensitivity to toxic substances and because they are a widely accepted model for the evaluation of cytotoxicity of biomaterials. HeLa cells were cultured in Dulbecco's Modified Eagle Medium (DMEM) supplemented with 10% foetal bovine serum (FBS, Gibco, USA) at 37 °C with 5% CO_2_ in a humidified incubator. Cells were seeded in 96-well tissue culture plates at 1 × 10^5^ cells per mL density prior to the experiment and incubated overnight for cell attachment and stabilisation. Fabric samples coated with ZnO were sterilised by UV irradiation for 30 min and then immersed in 2 mL of cell culture medium. The suspensions were vortexed well and incubated for 6 h to generate fabric-conditioned media. After the conditioning period, the culture medium in the cell plates was aspirated and the cells were gently washed once with sterile Hank's Balanced Salt Solution (HBSS). Then, 0.1 mL of the conditioned medium with the ZnO-coated fabric samples was added to each well. Cells were then incubated in a humidified atmosphere containing 5% CO_2_ at 37 °C for 24 h. Cells cultured in standard growth medium without ZnO-coated fabrics served as the negative control (untreated group), while cells exposed to medium containing 0.01% Triton X-100, a standard cell lysis agent, served as the positive control. After 24 h of exposure, cellular morphology was examined using a Nikon Eclipse TE2000 phase-contrast microscope to assess any treatment-induced morphological changes. Following microscopic observation, the culture medium was carefully removed and replaced with 50 µL of MTT solution (5 mg mL^−1^ prepared in DMEM). The plates were wrapped in aluminium foil and incubated in the dark for 4 h to allow viable cells to reduce MTT into insoluble purple formazan crystals. After incubation, formazan crystals were dissolved by adding 100 µL of dimethyl sulfoxide (DMSO) to each well. Blank wells containing DMEM, MTT solution, and DMSO, but no cells, were included to correct for background absorbance. The absorbance of developed solutions was spectrophotometrically measured at 570 nm with background correction at 630 nm. The cell viability was expressed relative to the untreated control group, which was set at 100%. The viability of the cells treated with the ZnO-coated fabric samples was then normalised to this value and expressed as a percentage of the control.

### 2.16 Thermal management

The thermal cooling ability of the ZnO-coated fabrics was demonstrated by cutting the coated and uncoated samples into 4 × 2 cm^2^ and placing them over the palm. The samples were held in the hand for 30 seconds and then the temperature was measured with the thermal camera.

## 3 Results and discussion

### 3.1 ALD of ZnO nanocoating on cotton fabric

**Fig. 1:**
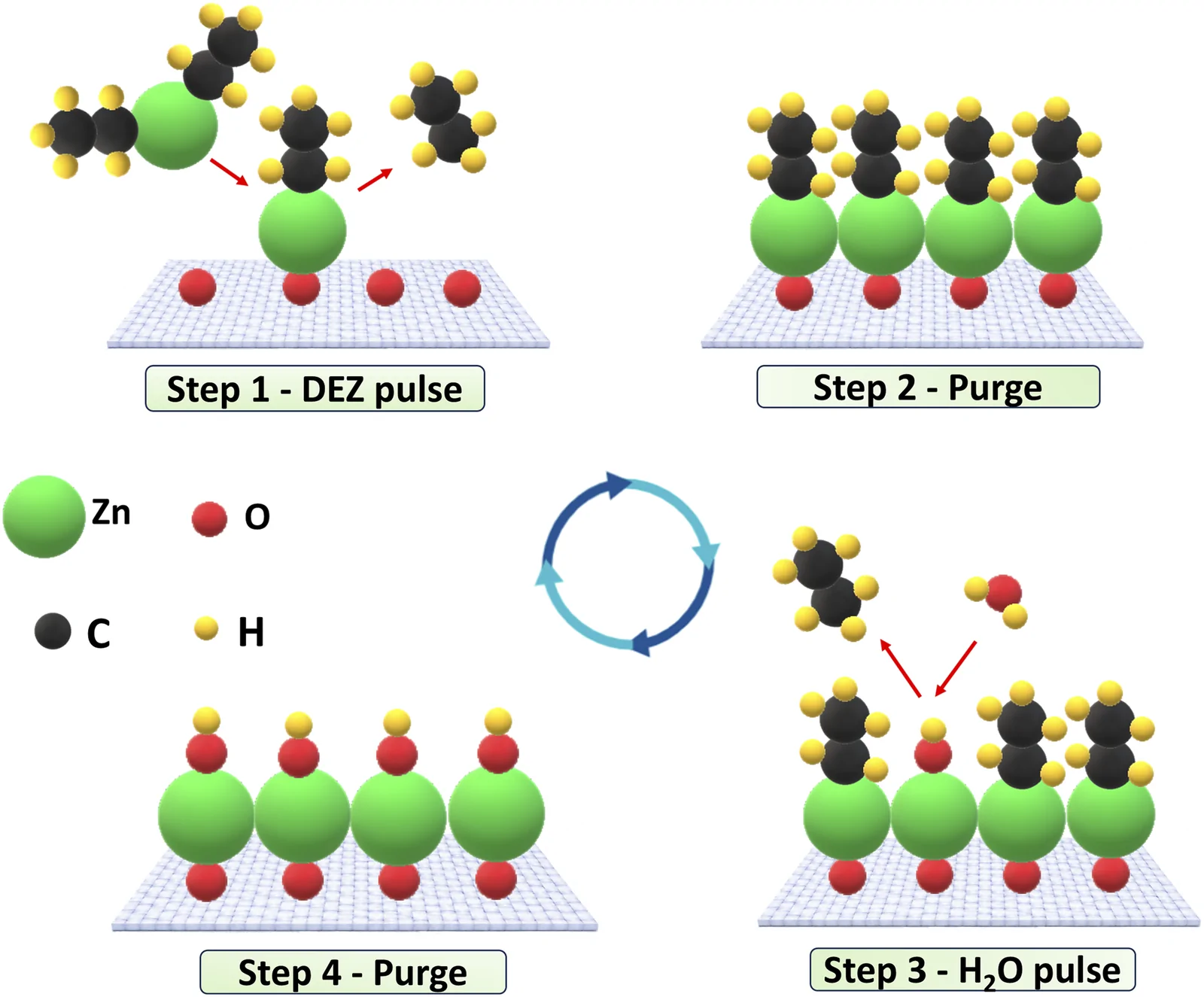
Schematic illustration of the thermal ALD growth mechanism of ZnO on cotton fiber surfaces using diethylzinc (DEZ) and H_2_O precursors.

The schematic of the ALD deposition is presented in Fig. 1. In the 1st step, a pulse of diethylzinc (DEZ) was introduced into the ALD reaction chamber. The cotton substrate contains hydroxyl (–OH) groups onto which DEZ molecules adsorb.^22^ This is a ligand–exchange reaction, where one of the ethyl groups of DEZ is transferred to the hydroxyl (–OH) group to form a monoethylzinc intermediate group and gaseous ethane. The reaction becomes self-limiting once all available surface hydroxyl groups have reacted.

1 Surface–OH + Zn(C_2_H_5_)_2_ → Surface–O–ZnC_2_H_5_ + C_2_H_6_↑

In the 2nd step, the reaction chamber was purged with an inert gas N_2_, for 30 seconds to remove all the excess unreacted DEZ. In step 3, water vapor reacts with surface-bound ethylzinc groups.

2 Surface–O–ZnC_2_H_5_ + H_2_O → Surface–O–ZnOH + C_2_H_6_↑

The subsequent removal of H_2_O vapor at step 4 from the reaction chamber resulted in the formation of ZnO coating on the textile surface.

### 3.2 Morphological analysis

The morphological and elemental compositions of the uncoated and coated cotton samples were examined using SEM coupled with energy-dispersive EDX. Fig. S1a and e show the surface morphology of the uncoated cotton sample and a single fiber, with a highly fibrous and rough surface, typical of cellulose fibres.^24^ Elemental mapping of the uncoated samples showed a uniform distribution of carbon and oxygen consistent with cellulosic fibres (Fig. S1b, c, f and g).^25^ As expected for the uncoated cotton, the presence of ZnO was not detected by EDX (Fig. S1d and h).

**Fig. 2:**
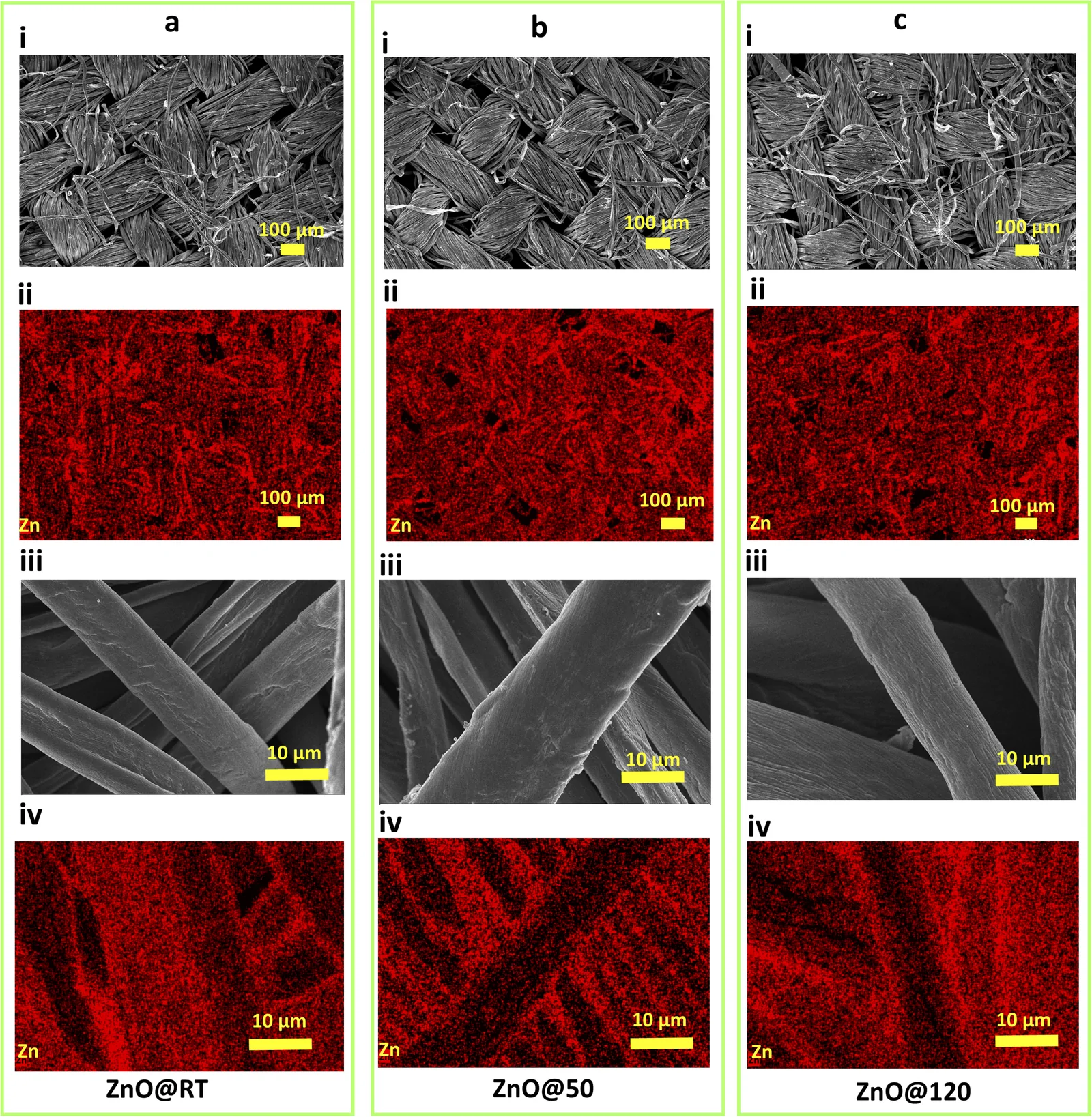
SEM morphology and Zn elemental mapping of ZnO-coated cotton fabrics deposited at different ALD temperatures. (a–c-(i)) Low-magnification SEM images and (a–c-(ii)) corresponding elemental mapping of the cotton woven structure at room temperature, 50 °C, and 120 °C, respectively. (a–c-(iii)) High-magnification SEM images of individual coated fibers and (a–c-(iv)) corresponding high-resolution Zn-mapping.

Substantial change in surface morphology and elemental composition was observed after the pristine cotton was coated with ZnO through the ALD deposition process. SEM analysis was also conducted to understand the morphology of the coated samples (Fig. 2a(i)–c(i)). As demonstrated by elemental analysis, all coated samples exhibited a relatively uniform coating distribution of ZnO (Fig. 2a(ii)–c(ii)). SEM EDX analysis also clearly showed a tiny pinhole-like space between multiple weaves. These empty spaces can be eliminated by using a very tightly bound woven structure; however, for design and user comfort purposes, most woven fabrics consist of these tiny spaces in between their structure. It is also clear that ALD provides a conformal ZnO coating on fibers, covering only the fiber surface without blocking or filling the open spaces in the fabric structure. Unlike other coating methods, ALD largely preserves breathability while causing only a modest reduction in air permeability.

Then, single-fibre SEM analysis was performed for the coated samples. The SEM image of ZnO@RT showed a slight roughening and sporadic particulate features (Fig. 2a(iii and iv)). Thus, the limited surface diffusion of precursor molecules at low temperatures, leading to incomplete coalescence of the film, can be attributed to the increased roughness. This condition also led to a slightly weak Zn signal in the EDX spectra (Fig. S2a). The intermediate deposition temperature (ZnO@50) showed increased nucleation and growth of ZnO as also indicated by a stronger Zn signal in EDX spectra (Fig. 2b(iii, iv) and S2b). The higher temperature deposition (ZnO@120) helped a bit in getting a smoother ZnO coating along with complete coverage of cotton fibre with ZnO and a higher Zn signal in EDX elemental analysis, indicating successful growth of the ZnO film (Fig. 2c(iii, iv) and S2c).

The washing durability of the coated samples was also investigated by SEM-EDX quantitative analysis after accelerated washing cycles. The EDX analysis confirmed the uniformity of the ZnO coating even after 5 washing cycles (Fig. S3). The quantitative analysis showed a decrease in ZnO content over the 5 cycles. However, the decrease in ZnO content with washing started to saturate upon increasing the number of washing cycles for all samples, indicating the durability of the washing process for the coated samples (Table S3).

### 3.3 Structural analysis

**Fig. 3:**
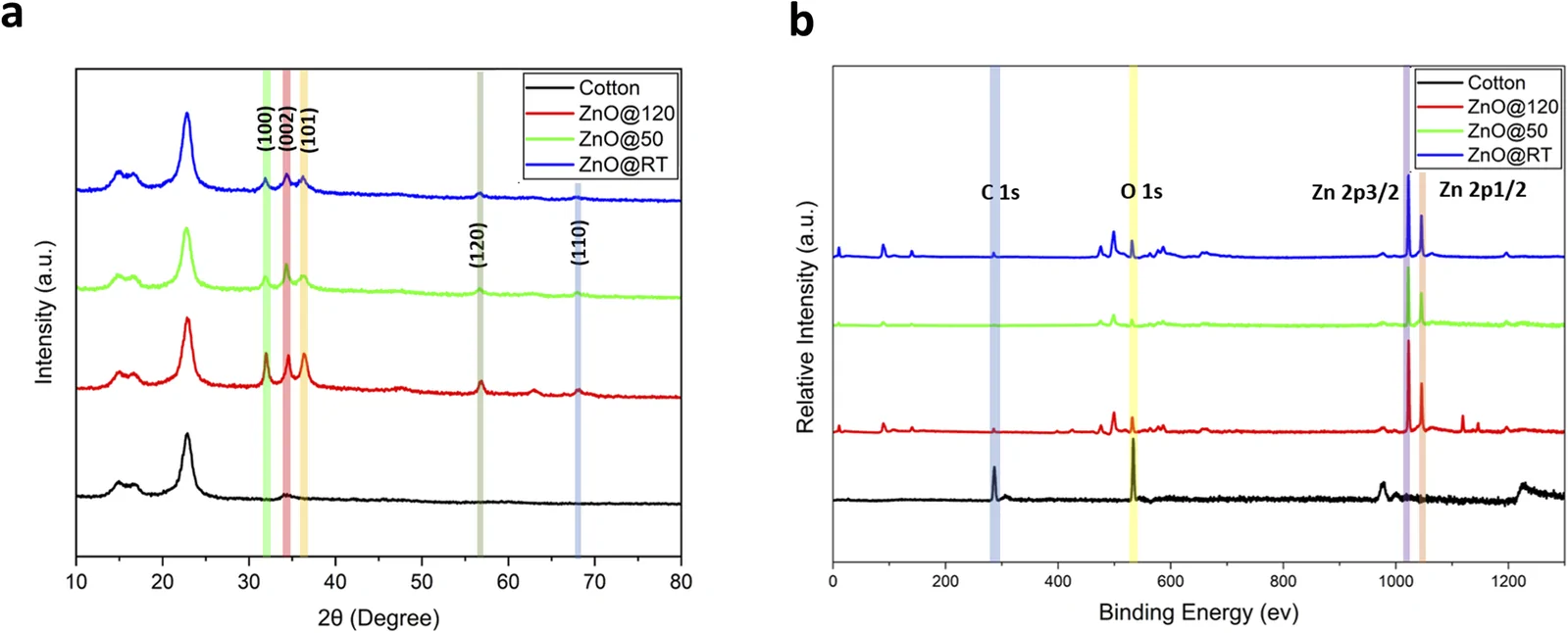
Structural and surface chemical analysis of uncoated and ZnO-coated cotton fabrics deposited at different ALD temperatures. (a) XRD patterns demonstrating characteristic ZnO wurtzite reflections. (b) XPS survey spectra of uncoated and coated samples, highlighting the emergence of Zn 2p_3/2_ and Zn 2p_1/2_ peaks.

XRD is a technique that determines the diffraction pattern of X-rays incident on a sample for determination of the crystalline structure, phase composition, and preferential orientation of a material. The structural evolution of the ALD process of fabric coated with ZnO is characterised by XRD analysis of the coated and uncoated samples (Fig. 3a). The XRD spectra revealed a broad peak at 22.8° for both the coated and uncoated samples corresponding to the crystalline phase of cellulose Iβ.^25^ The XRD pattern of the coated samples showed diffraction angles of 2*θ* ≈ 31.7° (100), 34.4° (002), 36.4° (101), 56.8° (102), and 68° (110), which correspond to the hexagonal wurtzite ZnO lattice structure.^26^ With an increase in the deposition temperature, a systematic enhancement in the peak intensity and the sharpness associated with ZnO was observed, indicating improved crystallinity at high temperature (ZnO@120). A much broader and less intense peak was detected for ZnO@50 and ZnO@RT samples, which supported the evidence of forming smaller crystallite sizes or greater structural disorder. The phase purity of the formed ZnO was confirmed by the absence of any other impurity phase in the XRD spectra. As expected, the ZnO peaks were not present in the uncoated cotton sample, which confirmed its pristine nature. The results clearly confirm the conformal coating of crystalline ZnO on the cotton substrate, and the crystalline quality is positively correlated with the increased ALD process temperature.

### 3.4 Chemical-state analysis

The surface chemistry and deposition behaviour of the coated and uncoated samples were characterised by XPS analysis (Fig. 3b). The survey on the uncoated cotton sample showed that the cellulose backbone of cotton leads to the formation of carbon and oxygen species. The uncoated cotton showed a dominant C 1s peak at 285 eV and a sharp O 1s peak at 531.5 eV, which is due to the hydroxyl-rich environment of the cellulose chain.^25^ The ZnO deposition resulted in a significant reduction of the C 1s and O 1s peaks, which were replaced by Zn 2p signals, Zn 2p_3/2_ peak (1022 eV) and Zn 2p_1/2_ peak (1045 eV),^27^ attributed to the ZnO coating that obscured the bare cotton functionalities.

**Fig. 4:**
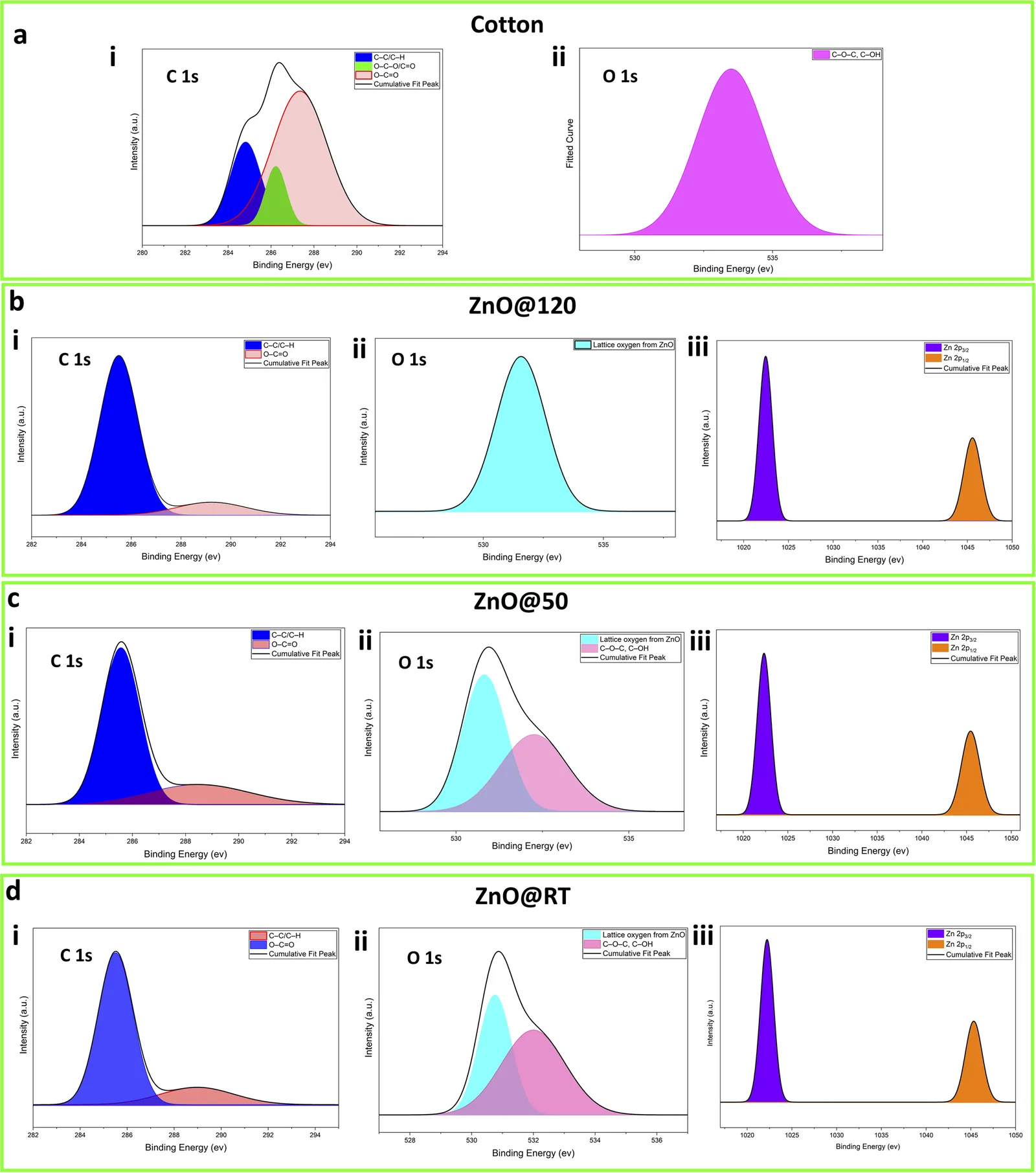
High-resolution XPS spectra of uncoated and ZnO-coated cotton fabrics. a (i and ii): C 1s and O 1s signals from uncoated cotton. b (i and iii): XPS HR spectra for ALD coating at 120 °C. c (i and iii): XPS HR spectra for ALD coating at 50 °C. d (i and iii): XPS HR spectra for ALD coating at RT.

The high-resolution C 1s spectrum of the uncoated cotton showed the oxygenated carbon environment with three distinct peaks, as shown in Fig. 4a(i). The 1st peak is observed at 284.8 eV and is assigned to C–C/C–H bonds. A second main component was found at about 286.2 eV and can be assigned to C–O bonds characteristic of ether and hydroxyl functionalities in cellulose. The third peak at around 288.5 eV can be assigned to O–C=O species, indicating the presence of carboxyl or ester groups formed due to surface oxidation or exposure to the environment. These results are in agreement with the surface chemistry of untreated cotton fibre, clearly dominated by C–C/C–H and C–O bonds, with a minor contribution of oxidised carbon species.^25^ The high-resolution C 1s XPS spectra of ZnO@120/50/RT samples shown in Fig. 4b(i)–d(i) showed a change in surface chemistry as compared to the uncoated cotton fabric sample. The coated samples were mainly dominated by the C–C/C–H bonds at a binding energy of 284.8 eV.^27^ A less intense orbital peak at 288.5 eV was also observed and attributed to O–C=O species. The absence of C–O groups was observed, which is a native characteristic of cellulose samples.^27^ This suppression is due to the conformal ZnO overlayer that masked the hydroxyl (–OH) and ether (C–O–C) functionalities singled out by the surface-sensitive nature of XPS.^28^ These results indicated successful coverage of the cotton substrate with ZnO.

The O 1s spectra may be further evidence of the formation of ZnO. The deconvoluted O 1s spectra of the uncoated cotton in Fig. 4a(ii) showed a single peak corresponding to surface hydroxyl groups and glycosidic linkages (531.5 eV).^25^ However, ZnO@120/50/RT samples exhibited multiple components, including lattice oxygen in Zn–O bonds (530.0 eV) and surface hydroxyl groups (531.5 eV) (Fig. 4b(ii)–d(ii)). The intensity of Zn lattice oxygen peaks increased with the deposition temperature, which indicated the significant effect of thermal conditions on precursor adsorption and film nucleation during the high-temperature ALD process.^26^ In the case of ZnO@120, ZnO growth was clearly favoured, giving rise to a sharp Zn lattice oxygen single signal, while only weak Zn lattice oxygen peaks were detected for the ZnO@RT sample. Interestingly, the O 1s spectrum of ZnO@120 did not exhibit any obvious hydroxyl-related peak, which was a striking difference from the ZnO@50 and ZnO@RT samples. This clearly showed that a higher deposition temperature resulted in more complete precursor reactions and the formation of a continuous, stoichiometric ZnO layer with minimal surface hydroxylation. Low-temperature coatings, however, have significant hydroxyl and C–O–C contributions, indicating impurities and residual substrate contributions.^26^

Fig. 4b(iii)–d(iii) for ZnO@120/50/RT samples also indicate the deposition of ZnO on the cotton surface, as shown by the appearance of the distinct Zn 2p_3/2_ (1022 eV) and Zn 2p_1/2_ (1045 eV) peaks, which proved the incorporation of Zn^2+^ species on the cotton surface.^27^ The good agreement of Zn 2p binding energies with the literature confirmed the deposition of stoichiometric ZnO and showed that no metallic Zn or other Zn compounds were deposited during the ALD process. The XPS analysis clearly demonstrated the correlation between the quality of the ZnO coating and processing temperature. These insights not only validate the successful integration of inorganic ZnO with organic cotton substrates but also highlight the importance of deposition temperature in tailoring surface chemistry and functionality for advanced textile applications.

### 3.5 Thermal analysis

DSC measures the heat flow into or out of a material as a function of temperature. It is used for the thermal transition detection of materials such as melting, crystallisation, and decomposition. All the samples exhibited a distinct endothermic peak in the range of 320–370 °C (Fig. S4a), which can be ascribed to the glycosidic linkage cleavage in cellulose. At the temperature of degradation, the cellulose backbone of cotton was pyrolytically cleaved, breaking the glycosidic bonds to yield volatile products (such as levoglucosan, tar, CO_2,_ and other low-molecular-weight gases) and a carbonaceous char.^29^ The uncoated cotton had the lowest decomposition temperature and the largest magnitude of the decomposition, indicating a less thermally stable structure. Conversely, all the ZnO-coated samples showed a right shift to a higher temperature, and the highest temperature and the most attenuated peak were observed for the ZnO@120 sample.^30^ This result indicated a progressive improvement of the thermal stability with ZnO-coated fabrics, where a high-quality and homogeneous ZnO coating acted as a protective barrier, limiting oxygen flow and mass transfer retarding heat flow and raising the degradation temperature.^31^

TGA monitors the change in mass of the sample as the temperature changes, providing data on moisture loss, decomposition, and the formation of residual char or inorganic content. Samples displayed a significant weight loss at 300 °C and 400 °C, owing to the pyrolytic decomposition of cellulose (Fig. S4b).^30^ Uncoated cotton showed the greatest weight loss and least residue (2.5% residue of original weight at 600 °C). Coated samples showed considerably more residue due to retention of the inorganic ZnO fraction.^29^ The percentage of residue of ZnO@120 (19.3%) at 600 °C was higher than that of the ZnO@50/RT samples (17.8% and 14.9%, respectively). This result can be correlated with the quantitative SEM-EDX analysis (Table S3), which showed higher deposition of ZnO in ZnO@120 than in the ZnO@50 and ZnO@RT samples.

### 3.6 Mechanical testing

**Fig. 5:**
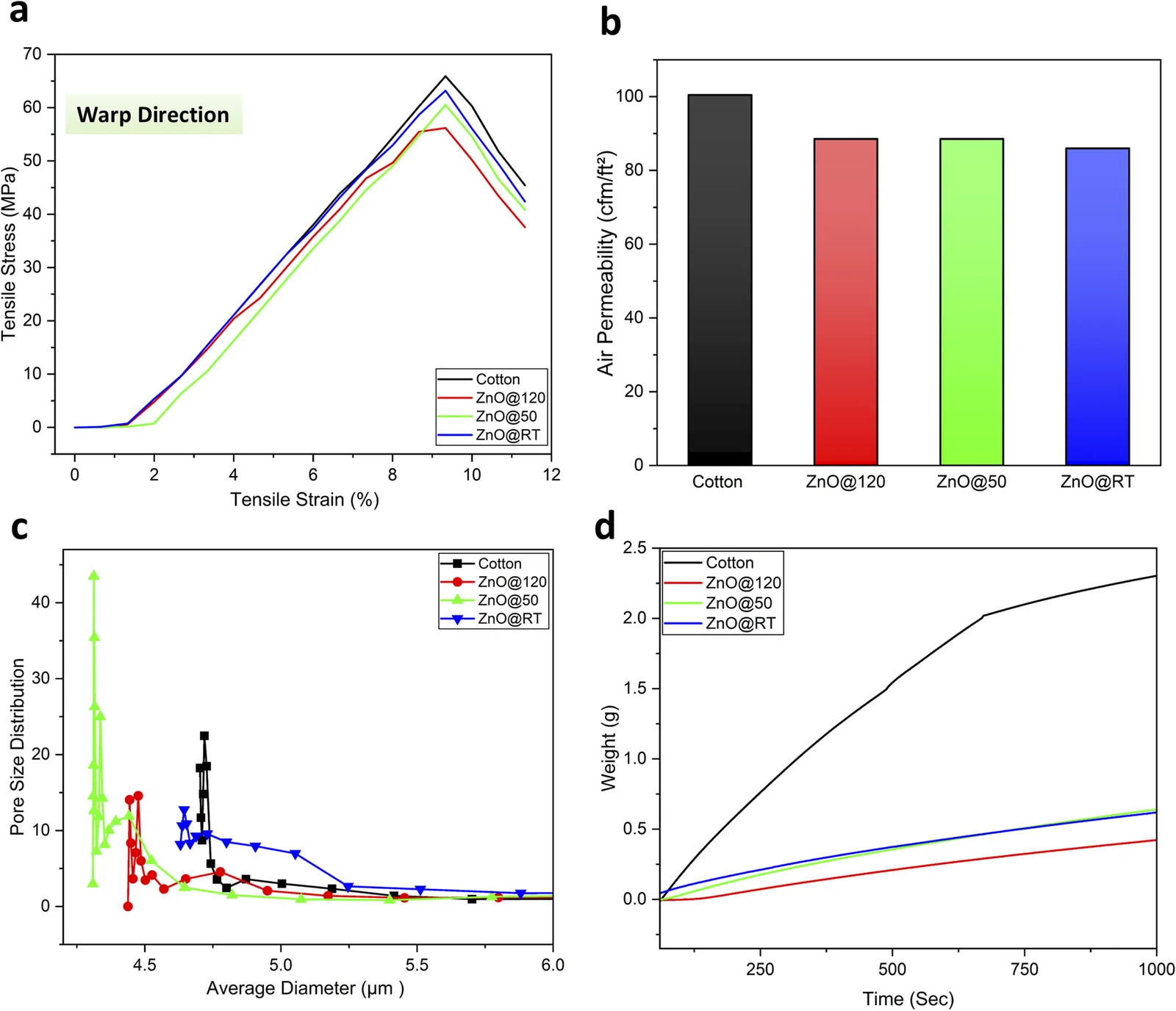
Mechanical and textile performance of uncoated and ZnO-coated cotton fabrics. (a) Tensile stress–strain test, (b) air permeability test, (c) pore size distribution, and (d) water absorbency test for the fabric samples.

Fig. 5a and S5 present the tensile stress–strain curves for uncoated and coated cotton samples in warp and weft directions, respectively. The tensile strength of the coated samples was very similar to the original tensile strength of the uncoated cotton fabric. But the coated samples had a slightly lower tensile strength. This is often reported for functional nanocoating, which can increase surface stiffness, limit fibre slippage, or increase local stress concentrations. However, the reduction was very little for ZnO-coated samples, and thus their ability to be used as a functional fabric for desired textile performance was retained. Overall, the warp direction performed better, with uncoated cotton reaching a maximum tensile strength of 66 MPa at approximately 10% strain (Fig. 5a). All coated samples showed a slight decrease in tensile strength after deposition, with ZnO@RT being closest to uncoated cotton, while the higher deposition temperatures (ZnO@50 and ZnO@120) lowered the peak tensile properties. The same trend was observed in the warp direction. Again, the highest tensile peak was obtained for uncoated cotton (around 11 MPa) (Fig. S5). The coated samples showed a slight decrease in the maximum stress and elongation also in the weft direction. A decrease in the total tensile strength in the weft direction was observed for the coated and the uncoated structures. This was caused by the weft yarn's yarn density, the alignment of the fibres, and the construction properties of the fabric. The warp yarns are denser and more aligned parallel to the length of the fabric, which generally has a higher load-bearing capacity. In contrast, the weft yarns are less packed, and fibre slippage is often observed during the stretching process, which affects the tensile strength properties.^32^

Martindale abrasion testing was performed to determine fabric resistance to surface wear and mechanical damage from repeated rubbing in normal use. It assesses the endurance of textile coatings and their ability to keep their function under real service conditions. Fig. S6 shows SEM images and the corresponding Zn elemental mapping after 500 Martindale abrasion cycles. The woven structure remained intact after repeated abrasion, and the coating of ZnO still conformed to the cotton fibres without significant peeling or delamination. The higher-magnification SEM (in the inset) shows the retention of the ZnO layer on the fibre surfaces in all three coated samples, with slight roughening and localised wear due to mechanical friction during the abrasion. Also, EDX elemental mapping shows a uniform distribution of Zn signals over the fabric surface, confirming the retention of the ZnO coating after the abrasion test. The Zn signal is persistently present, showing good interfacial adhesion between the ALD-grown ZnO and the cotton substrate, which is a major benefit of atomic layer deposition over conventional coating methods.

### 3.7 Air permeability and moisture absorbency

Air permeability is an important comfort parameter of a fabric, which measures how easily air can pass through a fabric. The air permeability test on uncoated cotton showed higher air permeability, whereas ZnO-coated samples showed a slight decrease in air permeability. This can be due to the presence of the ZnO coating on the fibre surface that could cover some of the interlayer spacing and provide additional resistance to airflow.^33^ However, ALD-based coating provides maximum breathability to the fabric compared to other textile coating techniques such as sol–gel, dip coating, or resin lamination.^34^ The air permeability of the uncoated cotton was found to be 100.44 cfm ft^−2^, and the coated samples showed air permeability in the range of 86–88 cfm ft^−2^ (Fig. 5b). The conformal coating on the cotton fabric resulted in most of the fabric pores remaining open even though the air permeability decreased slightly. This assumption is also supported by the fabric porosity testing in Fig. 5c and S7, where the effect of ZnO coating on the pore size distribution is compared. The bare cotton exhibited a distribution peak of 4.7 µm, while the coated samples showed a slight decrease in the average pore diameter, resulting in a slight decrease in the air permeability.

The M/K GATS is a device used for measuring water absorbency and wicking, utilizing zero-pressure capillary uptake and gravimetric measurement. The water-absorbency behaviour of different uncoated and coated samples, measured as weight gain over time due to water absorption, is shown in Fig. 5d. Uncoated cotton showed a very high water intake and exceeded 2 g after a test duration of 1000 s, which is attributed to the presence of numerous surface hydroxyl (–OH) groups and the porous structure of the original cellulose fibres, which attract and retain water *via* hydrogen bonding and capillary action.^35^ Conversely, the water absorbency decreased for coated samples. The ZnO@120 sample exhibited the least absorbency, while the ZnO@50 and ZnO@RT coated samples exhibited slightly higher absorbency. This result indicates that the deposition of ZnO layer can reduce the surface hydrophilic properties due to the masking of cotton's natural hydrophilic surface groups.^36^ The M/K GATS results therefore demonstrate that ZnO functionalization introduced a trade-off between the added multifunctional properties and the intrinsic liquid moisture-management capability of cotton. The reduced liquid uptake of the ZnO-coated fabrics represents a limitation for applications requiring efficient sweat transport and prolonged next-to-skin comfort, particularly activewear and sportswear, where rapid wicking and moisture removal are essential. However, this limitation might be less important for protective and functional textiles where the focus is being placed on antibacterial activity, UV protection, and self-cleaning performance rather than rapid liquid moisture transport.

### 3.8 Color spectroscopy

Color spectroscopy results in Fig. S8 quantified the change in color of cotton fabric with CIELab color coordinates before and after ALD of ZnO nanocoating. The three coordinates are L*, a*, and b* that can be described in CIE Lab colour space and provide a standardised way to represent colour. Chroma (C*) is the saturation or intensity of a colour and is a function of a* and b*, which are the quantities of the position in the colour space. L* is the lightness.^37^ Fig. S8a shows L*, a*, and b* values of the uncoated cotton sample, which was used as a reference to evaluate the coated samples. Among all the samples, the ZnO@120 sample exhibited the highest colour change compared to the uncoated sample, with a significant decrease in the L* value (94.14), implying the darkening of cotton fabric and becoming visually yellower than the uncoated cotton (Fig. S8(b, c) and S9). This behaviour can be explained by the thermal degradation of the cotton substrate during the ALD process. It is known that cotton fibres show a yellowish colouration when subjected to temperatures in the range of 120–140 °C.^38^ Since the ALD deposition was performed at 120 °C, the marked increase in yellowness observed for the sample ZnO@120 is most likely associated with the beginning of thermally induced degradation and oxidation reactions in the cotton fibres, rather than the ZnO coating itself. For the ZnO@50 sample, the value of L* decreased slightly while b* and C* rose relative to uncoated cotton, indicating a modest darkening and an increase in yellow saturation, though less pronounced than in the ZnO@120 sample. ZnO@RT demonstrated the slightest color change, which remained very similar to the uncoated cotton. The L* parameter of ZnO@RT (95.38) remained almost similar to that of uncoated cotton (95.41), indicating limited darkening, while b* and C* increased marginally, suggesting only a slight increase in yellowness and chroma. This result represents a trade-off between the quality of ZnO deposition and textile coloration of textile substrates, where higher temperature may favor crystalline ZnO formation on the textile but brings a slightly different shade to the substrate due to thermal degradation.

Overall, these results demonstrate a trade-off between ALD deposition temperature, coating characteristics, functional performance, and textile appearance. Although ZnO@120 exhibited higher ZnO crystallinity and more complete surface coverage, it also caused noticeable yellowing of the cotton fabric. In contrast, ZnO@50 exhibited moderate crystallinity without noticeable color change while providing functional performance comparable to that of ZnO@120 across the evaluated applications, including UV blocking, self-cleaning, and thermal performance, together with strong antibacterial activity (ref: sections 3.19, 3.10, and 3.11). Therefore, considering the balance between coating quality, multifunctional performance, and preservation of the original textile appearance, 50 °C ALD deposition may represent the preferred processing condition for most practical applications.

### 3.9 Photocatalytic self-cleaning

**Fig. 6:**
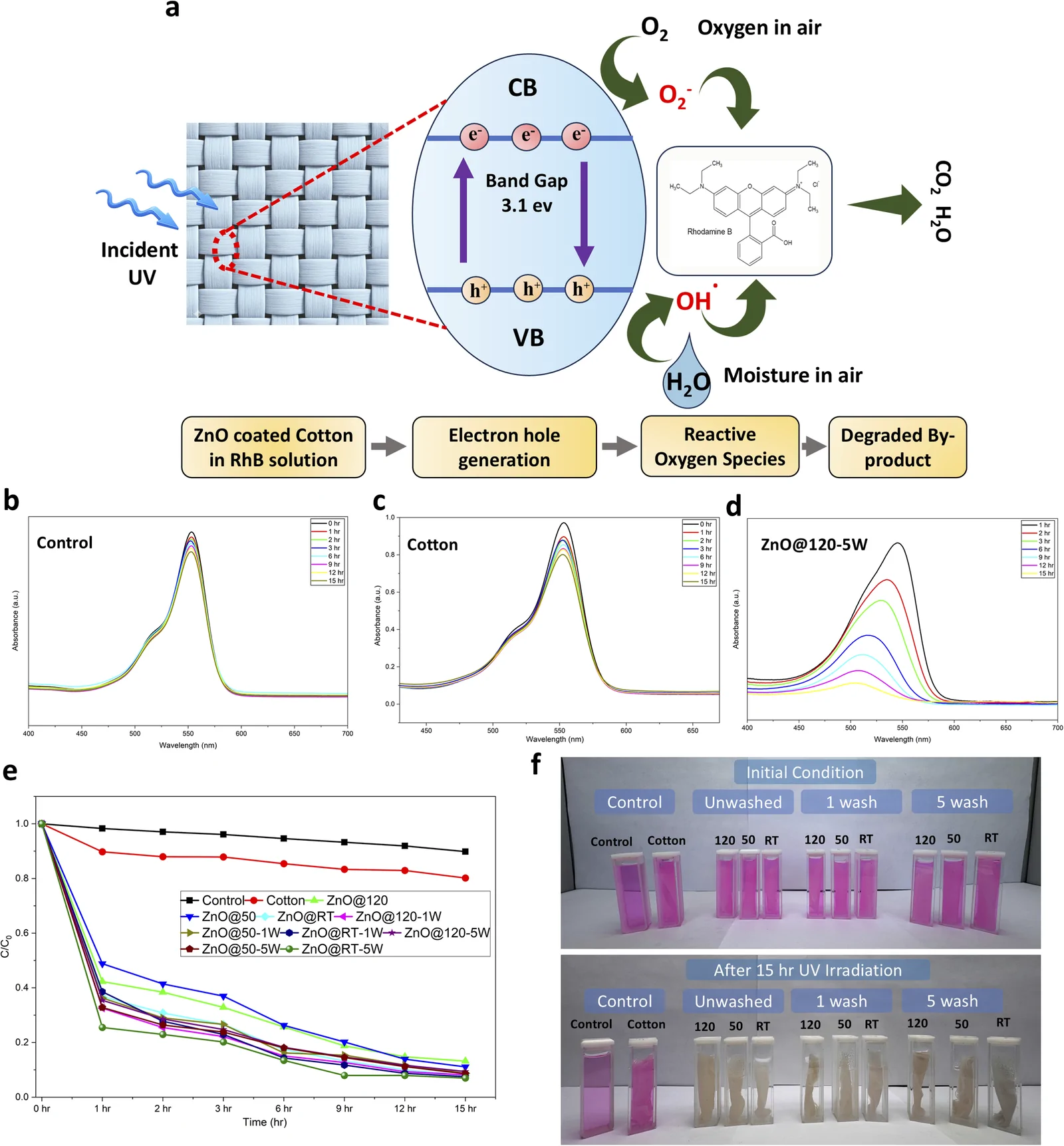
Photocatalytic self-cleaning performance of ZnO-coated cotton fabrics under 365 nm UV irradiation. (a) Mechanism of photocatalytic self-cleaning for ZnO coated fabric. (b–d) UV-vis absorbance spectra for control, cotton and ZnO@120-5W in RhB solution over 15 hours. (e) Relative RhB concentration (*C*_*t*_/*C*_0_) over time. (f) Photographs of RhB solution at the start and after 15 hours, illustrating dye degradation in ZnO-coated samples.

ZnO nanocoatings can provide photocatalytic self-cleaning ability by generating electron–hole pairs under UV light.^39^ When ZnO particles are exposed to solar/UV light (*hv*), electrons are excited from the valence band (VB) across the 3.1 eV band gap to the conduction band (CB), leaving positively charged holes (*h*^+^) in the VB. The photogenerated electrons in the CB react with dissolved oxygen to form superoxide radicals (O_2_^−^), and the holes oxidise surface water or hydroxide ions to generate hydroxyl radicals. These highly reactive oxygen species then attack the dye molecules, breaking the chromophore structure and ultimately converting the dye/organic dirt into harmless degradation products such as CO_2_ and H_2_O (Fig. 6a). Moreover, the ZnO surface is more hydroxylated, which enhances the hydrophilicity. Therefore, the combination of photocatalytic degradation and photoinduced wettability change makes the ZnO-coated surface effective for application as a self-cleaning textile.^40^

In this study, uncoated and ZnO-coated samples are immersed in Rhodamine (RhB) dye solution in a cuvette to test their photocatalytic efficiency under UV light. UV-vis spectroscopy was used to measure the absorption spectra before and after self-cleaning. The relative concentration of RhB is calculated as *C*_*t*_/*C*_0_ = *A*_*t*_/*A*_0_, where *C*_*t*_ and *A*_*t*_ represent the concentration of dye in the solution and the absorbance value at a specific time *t*, while *C*_0_ and *A*_0_ are the concentration and absorbance values under the initial condition, respectively. The ZnO@120-5W sample was immersed in RhB solution for 30 min before UV irradiation, in the absence of UV illumination. The absorbance peak was measured before and after immersion in solution. The result showed a prominent absorbance peak at around 554–557 nm for both test conditions with similar absorbance intensity (Fig. S10).^41^ The results show that ZnO@120-5W did not change the concentration and chemical structure of RhB before UV irradiation, even after 30 min of immersion. This clearly indicated that no chemical decomposition and minimal dye absorption took place during the immersion of fabric coated with ZnO into dye solutions. Thus, the same RhB concentration was maintained among all the samples during the 0th hr UV irradiation. Fig. 6b–d illustrates UV-vis absorption spectra of control, uncoated, and ZnO@120-5W samples of RhB solutions up to 15 h. Fig. 6e and S11 illustrate the subsequent relative change in concentration of the solutions containing coated and uncoated samples due to the UV illumination and their subsequent UV-vis absorbance spectroscopy source. The control (RhB solution only) and the uncoated sample show a slight decrease in peak intensity and concentration during 15 h UV irradiance, suggesting only minor degradation of the RhB solution by UV exposure. The solution with uncoated cotton showed a slightly higher loss in concentration of RhB, due to absorption of RhB into the cotton fabric. Conversely, all the ZnO-coated samples showed a drastic reduction in the absorbance peak, proving effective photocatalytic self-cleaning. The initial maximum photocatalytic reaction rate slowly saturated with the progress of the photocatalytic reaction which was due to the maximum initial dye concentration and maximum availability of active sites from the photocatalytic reaction. Although all coated samples demonstrated significant photocatalysis, some non-uniformity was observed in the rate of photocatalysis for the coated samples. In general, the washed samples possessed slightly better photocatalytic self-cleaning properties than the unwashed ones. However, ZnO@RT exhibited somewhat higher efficiency compared with the ZnO@120 and ZnO@50 cotton samples.

The improved self-cleaning performance after 1W and 5W cycles may be attributed to the partial rupture or microcracking of the ZnO coating layer as seen in the SEM images (Fig. S12). Such microcracks could expose fresh ZnO surfaces and provide more active sites for photocatalytic reactions. Washing may also remove loosely attached impurities/surface residues, which allows better contact of dye molecules, light, and water/oxygen with the ZnO layer.^42^ Also, a ruptured coating can increase surface roughness and the effective surface area, thereby improving light scattering and enhancing dye adsorption on coated fibres. It should be mentioned that the improved photocatalytic performance after washing may be a transient effect of the surface state but not a permanent enhancement of the photocatalytic self-cleaning. The formation of microcracks may initially generate fresh surfaces of ZnO and increase the availability of photocatalytically active sites. However, prolonged laundering may subject the ZnO coating to sustained mechanical stresses that may facilitate microcrack propagation and coalescence, possibly leading to local fragmentation or delamination of the ZnO coating and eventual degradation of its functional performance.

Fig. 6f and S13 present a visual comparison of the photocatalytic self-cleaning effect for RhB dye solutions treated with different cotton fabric samples over a 15-hour UV irradiation period. All cuvettes, including the control (RhB solution only), uncoated cotton and ZnO-coated cotton fabrics at different washing cycles, initially showed a uniform pink colour, indicating the uniformity of RhB concentrations and negligible absorption or degradation before irradiation. After 15 h of UV irradiation, the control and the uncoated cotton containing solution showed negligible change in RhB solution colour, which means no photolytic degradation and negligible dye removal by absorption. In contrast, the cuvette with ZnO-coated samples became completely transparent, which indicates almost complete degradation of the RhB dye pollutant due to photocatalysis. The fabric staining with RhB before and after photocatalysis is illustrated in Fig. S14. After 15-hour immersion in RhB solution, the uncoated fabric was completely stained by the pink dye. In contrast, the ZnO-coated cotton samples retained their original colour almost unchanged after immersing in the dye bath for 15 hours, which shows their strong photocatalytic self-cleaning ability.

### 3.10 UV protection

**Fig. 7:**
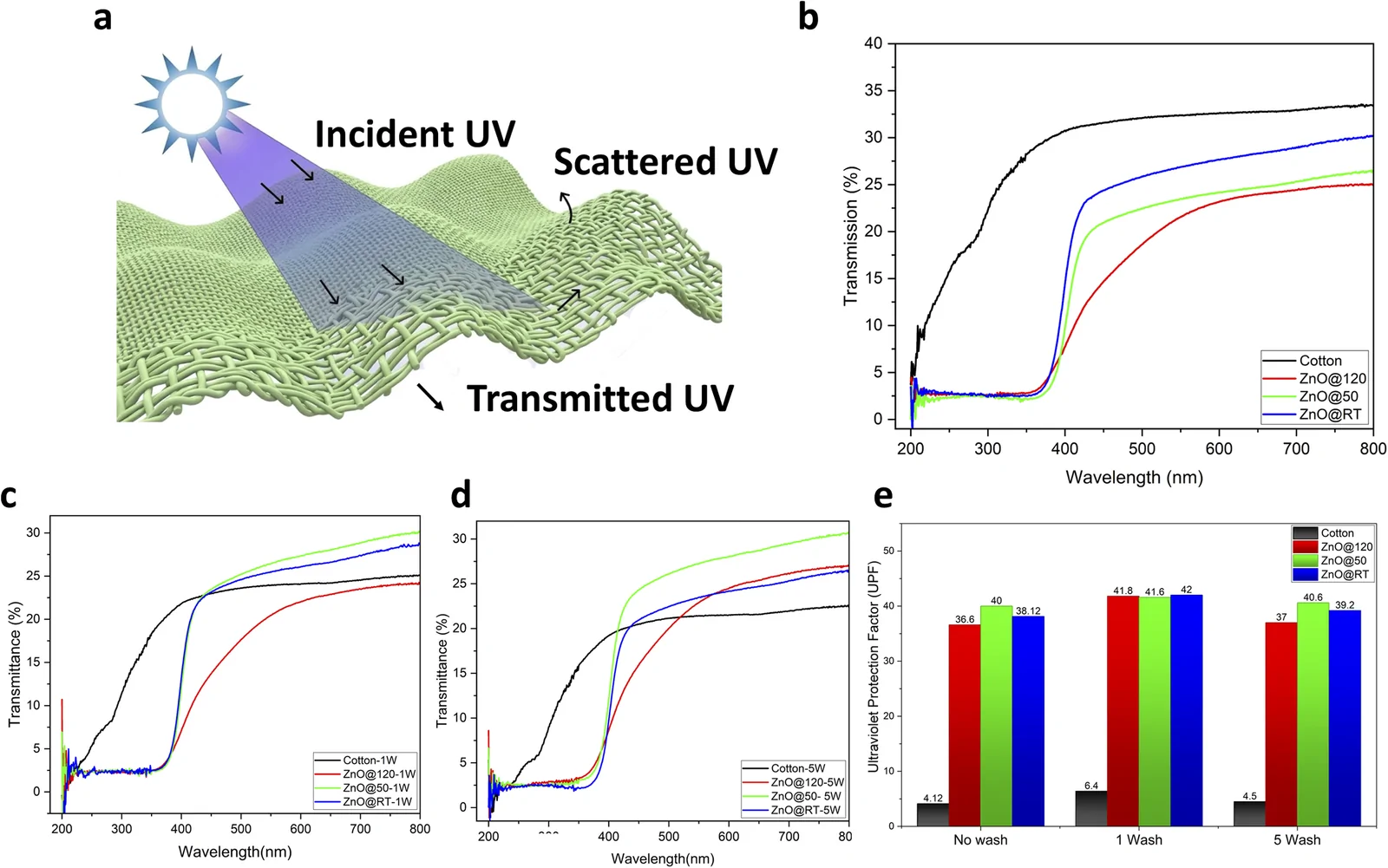
UV blocking test of the woven cotton. (a) Schematic of UV-blocking by ZnO-coated fabric. (b–d) UV-vis transmittance spectra of uncoated and ZnO-coated fabrics under different washing conditions. (e) Ultraviolet Protection Factor (UPF) of coated and uncoated fabrics before washing, after 1 wash, and after 5 washes.

ZnO thin coating is a good candidate for UV protection because of its optical and semiconducting properties. ZnO has a large band gap and thus strongly absorbs in the UVA (320–400 nm) and UVB (290–320 nm) regions of the UV spectrum but is relatively transparent to visible light. When a ZnO-coated surface is exposed to UV light, the electrons are either excited from the valence band to the conduction band or scattered. This double mechanism significantly decreases UV transmission and protects from harmful UV radiation (Fig. 7a). Thus, ZnO thin coatings on cotton are ideal for high performance UV protective clothing.^43^

The UV-vis transmittance results in Fig. S15 demonstrate that the uncoated textile substrates (cotton, nylon and polyester) have very different inherent UV-blocking capabilities due to fibre chemistry, fabric construction and fabric weight/thickness. Among the common textile substrates, polyester and nylon already showed relatively lower UV transmittance, indicating that these materials have better inherent UV-blocking capacity.^44–47^ Conversely, cotton showed significantly higher native UV transmittance, indicating a serious lack of UV protection, despite its wide use in everyday, healthcare and protective textile products. This makes the UV-blocking performance of cotton especially important. For this purpose, a lightweight 100 GSM woven cotton fabric was deliberately selected as a challenging substrate, as the lower fabric mass allows for higher UV penetration and offers a stringent platform to test the efficiency of ZnO coating.^23^ Therefore, the significant reduction in UV transmittance after ALD ZnO deposition will be of great importance, as it will show that the coating is able to compensate for the inherently weak UV protection of lightweight cotton and transform it into a much more effective UV-protective textile.

The transmission spectra of the uncoated and ZnO-coated cotton samples under different washing conditions are shown in Fig. 7b–d. The uncoated cotton showed much higher transmission in the UV region with an UPF value of 4.12 (Fig. 7e). The uncoated cotton transmits significant portions of UV radiation, but it does absorb some portions of UV radiation because of natural pigments, pectins, waxes, and minor amounts of lignin in the cotton fibre structure that act as UV-absorbing agents. This acts as a natural barrier to block UV transmission to a lower degree.^48^ However, the ZnO-coated sample exhibited a much higher UV shielding capability with the UPF factors in the range of 37–40 for the unwashed sample (Fig. 7e). Regardless of the deposition conditions, all coated samples demonstrated a transmission reduction close to 2.5%.

The uncoated and ZnO-coated cotton samples were tested for UV shielding stability against the laundry cycles of the fabrics by a washing test. The UPF surprisingly increased after the first washing step for both the ZnO-coated and uncoated samples. This may be due to the shrinkage of the woven structure that decreases the UV permeability for all the ZnO-coated and uncoated samples.^49,50^ Similar effects were also observed in previous studies where washing improved the UV protection of textiles. However, a slight decrease in the UPF was seen after the 5th wash cycle, where the UPF values again came very close to those at the pre-wash point. This may be due to the thinning of the fabric and partial damage/physical etching of the ZnO coating due to repeated washing of the fabric.

### 3.11 Antibacterial activity of ZnO-coated cotton

**Fig. 8:**
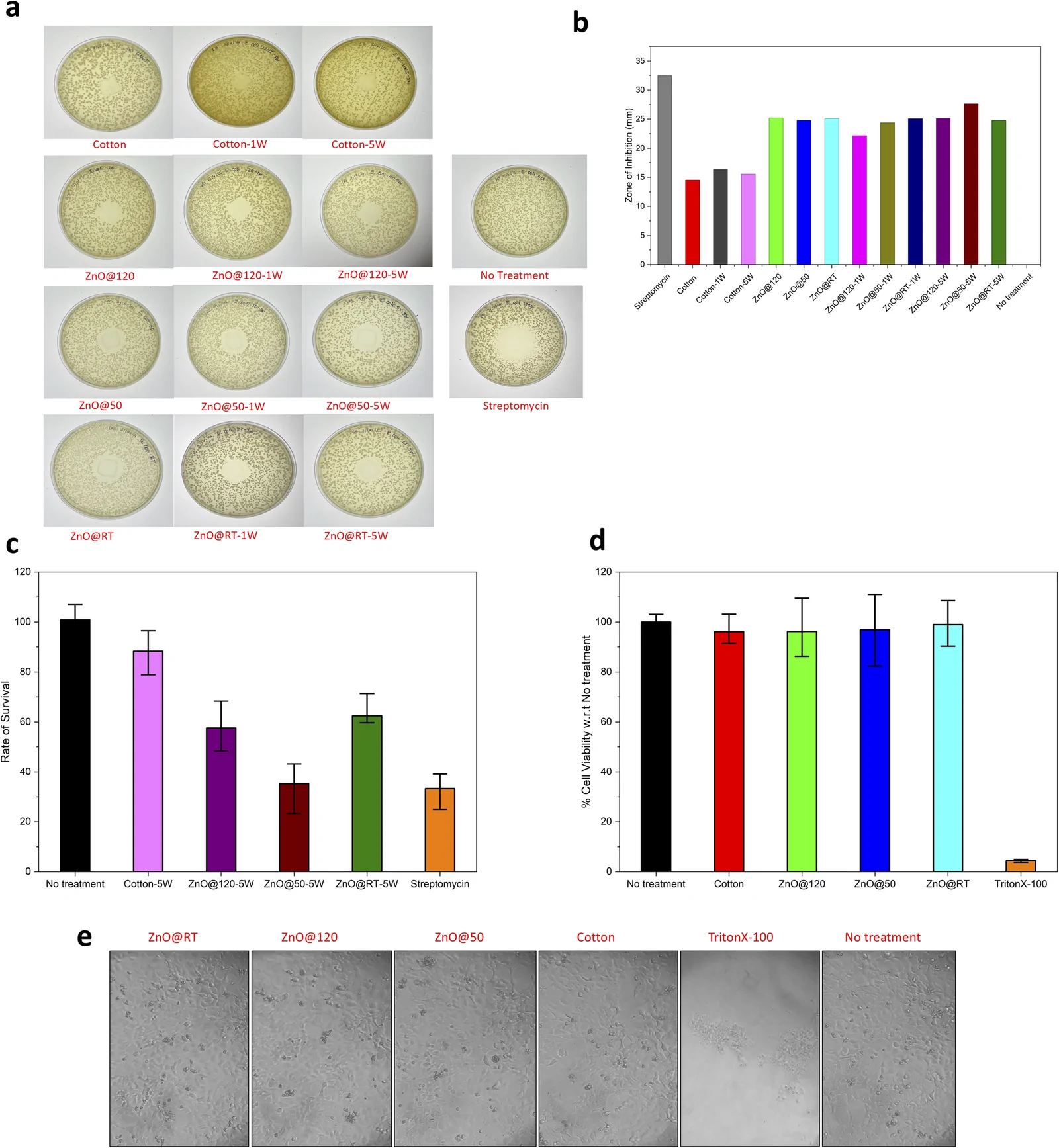
Antibacterial activity and cytocompatibility of ZnO-coated cotton fabrics. (a) Disk diffusion assay of uncoated and ZnO-coated cotton fabrics against *E. coli*. (b) Inhibition zone diameters of ZnO-coated fabrics. (c) Rate of bacterial survival after direct contact with samples for serial dilution assay. (d) Cell viability after exposure to fabric extracts. (e) Phase-contrast images of HeLa cells after 24 h of exposure.

The excellent antibacterial activity of ZnO is primarily attributed to the generation of reactive oxygen species (ROS) such as hydroxyl radicals and superoxide ions, leading to oxidative stress in bacterial cells. These oxygen species damage the cell membrane, proteins, and genetic material, leading to disruption of essential cellular functions and eventual bacterial death.^51^ The antibacterial activity of the ZnO-coated fabric samples was evaluated qualitatively by disc diffusion assay against *Staphylococcus aureus* (ATCC 25923, Gram-positive) and *Escherichia coli* (ATCC 25922, Gram-negative).^52^ The untreated disc did not show any zone of inhibition against either of the bacterial strains, confirming the absence of intrinsic antibacterial activity (Fig. 8a and S16a). Conversely, the ZnO@120, ZnO@50, and ZnO@RT samples demonstrated substantially greater zones compared to the uncoated cotton fabric. In particular, the ZnO@50 sample generated zones of 24.8 mm (unwashed) and 27.6 mm (5-wash). In contrast, uncoated cotton exhibited 14.5 mm zones against *E. coli* (Fig. 8b and Table S4). The highest antibacterial activity was displayed by the antibiotic positive control, streptomycin, with a zone of 32.5 mm. Notably, antibacterial activity was retained after several washing cycles, with 5-times washed samples showing similar or even improved activity compared to unwashed samples, suggesting the stability of the ZnO coating. A similar trend was observed for the coated samples against *S. aureus* (Fig. S16b), which can be attributed to the previously reported partial rupture or micro-cracking of the ZnO coating layer (Fig. S12), exposing a fresh ZnO layer for antibacterial activity. In addition, the washing process can create microcracks and expose more ZnO nanoparticles on the surface of the fabric. Therefore, ZnO can be more easily dissolved, and Zn^2+^ ions can be released, which can penetrate into the bacterial cells and cause antibacterial effects and membrane damage. This can be one of the reasons for the enhanced antibacterial activity of the washed samples.^53^

The antibacterial efficacy was evaluated quantitatively using 5-wash fabric samples, which represent the most stringent wash condition, by serial dilution assays against *E. coli*. Bacterial survival was expressed as a percentage of a no-treatment control (set at 100%) and compared to that of a streptomycin positive control. The no-treatment control showed full bacterial survival (100.8% ± 2.5%), confirming normal bacterial growth under assay conditions (Fig. 8c). The uncoated cotton-5W sample brought down the bacterial survival modestly to 88.3% ± 2.5%, consistent with its smaller zone of inhibition in the disc diffusion assay. In contrast, the ZnO@50-5W fabric reduced survival to 35.2% ± 3.0%, approaching the efficacy of streptomycin (33.3% ± 2.4%). ZnO@120-5W and ZnO@RT also showed meaningful antibacterial activity, reducing survival to 57.6% ± 3.3% and 62.5%, respectively. Together, these results establish that ZnO nanocoatings on cotton significantly reduce bacterial viability, with the ZnO@50-5W sample achieving near-antibiotic-level efficacy even after 5 washes.

### 3.12 Cytotoxicity of ZnO-coated cotton

The cytotoxicity of ZnO-coated cotton samples was quantified by indirect MTT assay on HeLa cells according to ISO 10993-555. In this assay, cell culture media were conditioned with fabric samples and applied to cells. The metabolic conversion of MTT to formazan by viable cells was measured spectrophotometrically and normalised to the no-treatment control (set at 100% viability). A positive cytotoxicity control was used to ensure assay sensitivity, consisting of Triton-X100, a cell lysis agent, which reduced viability to 4.4% ± 0.2% (Fig. 8d). All the ZnO-coated cotton samples showed high cell viability and no difference from the no-treatment control. The ZnO-coated cotton samples exhibited high cell viability similar to that of the no-treatment control. In particular, the viabilities for uncoated cotton fabric, Zn@RT, ZnO@50 and ZnO@120 were 96.1 ± 1.5, 99.0 ± 2.5, 96.9 ± 3.3 and 96.2 ± 2.7%, respectively. Overall, these results suggest that ZnO-coated cotton at all three ALD processing temperatures does not induce any measurable cytotoxicity to mammalian cells under the conditions tested.

The health and viability of HeLa cells treated with ZnO-coated cotton samples were visualised by phase contrast microscopy after 24 h treatment. The HeLa monolayer was maintained with a dense population of plump circular morphology, indicating the healthy condition of the cells even after 24 h exposure to the sample-containing media. Stress or detachment was not observed in any of the ZnO-coated samples, which was clearly observed in the cells treated with 0.1% Triton-X treatment, showing that the ZnO coated samples are non-hazardous (Fig. 8e).

In the antibacterial test, the bacterial cells are in direct contact with the ZnO-coated fibres, and the locally concentrated antibacterial effect is obtained through surface-mediated ROS generation, Zn^2+^ release and direct ZnO–bacteria interactions. The cytotoxicity test, however, uses an indirect conditioned-medium method, where the HeLa cells are not in direct contact with the ZnO-coated surface. Any Zn species released from the coating are distributed in the conditioning medium, whereas the short-lived surface-generated ROS may not survive at levels similar to those occurring locally at the bacteria–ZnO interface. These differences in exposure may therefore explain the significant antibacterial activity with high mammalian cell viability.

### 3.13 Thermal management

**Fig. 9:**
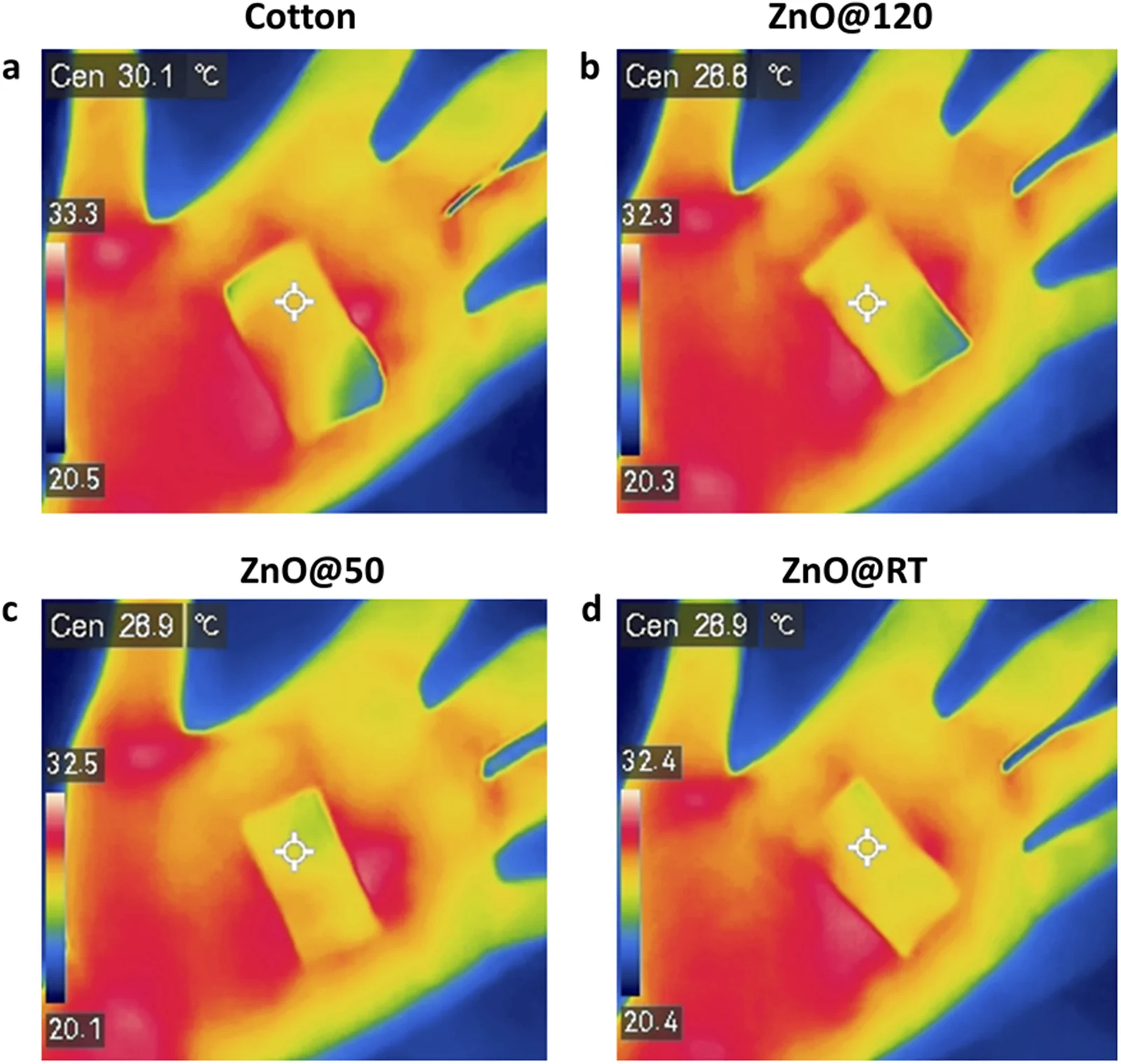
Thermal camera images comparing surface temperature of uncoated cotton and ZnO-coated cotton fabrics placed on the palm for 30 seconds. Uncoated cotton (∼30.1 °C) (a) exhibited higher temperature than ZnO-coated samples (∼28.8–28.9 °C) (b–d).

Fig. 9a shows that the surface temperature of the uncoated cotton sample was the highest and was recorded at 30.1 °C. Conversely, the temperature of the fabrics coated with ZnO was slightly lower. The temperatures of the coated samples were recorded as ZnO@120, 28.8 °C (Fig. 9b), ZnO@50, 28.9 °C (Fig. 9c), and ZnO@RT, 28.9 °C (Fig. 9d). The temperature difference between the ZnO-coated samples was negligible (≤0.1 °C), suggesting that the different coating conditions did not have a significant effect on the surface temperature. The increased temperature of the uncoated sample can be attributed to the porous nature of cotton fabric, which traps the air in its structure in a natural way and thereby makes the cotton fabric a poor conductor of heat. However, for the coated samples, the pores are partially filled, and the surface pores are covered by a coating of ZnO, resulting in low air entrapment and relatively higher thermal conductivity. This event may boost heat dissipation as heat is carried away from the skin to the environment more efficiently and avoid the build-up of unpleasant heat.^54^ Furthermore, the decreased surface temperature might also be attributed to the effect of the ZnO coating on the thermal-IR emissivity and radiative heat exchange of the cotton surface.^55^ The polar Zn–O lattice shows IR-active vibrational modes which are able to couple with the thermal radiation and therefore can help in radiative heat dissipation of the coated textile. Thus, the observed cooling could be explained by a mixture of conductive and radiative heat-transfer mechanisms.

## 4 Conclusion

The study showed nanoscale functionalization of cotton fabric by ALD of ZnO nanocoating. The study revealed the improved performance of ZnO-coated cotton fabric in photocatalytic self-cleaning, UV blocking, antibacterial and thermal comfort while retaining its vital mechanical and comfort textile properties. By adjusting the ALD temperature (RT, 50 °C, and 120 °C), we were able to establish well-defined correlations between coating morphology, crystallinity and multifunctional performance. The outstanding performance of ZnO nanocoating on cotton was evidenced by a photocatalytic self-cleaning effect ∼11 times higher, complete degradation of RhB dye within 15 h, and ∼10 times improved UV blocking with only <2.5% UV radiation transmitted. The ZnO-coated fabrics displayed long-term antibacterial activity against both Gram-negative and Gram-positive bacteria, with survival of *E. coli* decreased to 35.2%. The ZnO-coated fabrics showed excellent cytocompatibility with mammalian cell viability greater than 96% and no observable cellular damage. Although a full uniform ZnO coating was formed on the cotton surface, the coated sample still showed air permeability, tensile integrity, and wash durability, and the performance was stable after multiple washing cycles. The results are important for the integration of ALD into textile processing in general to form next generation functional textiles.

## Author contributions

Md Sazid Bin Sadeque: methodology, investigation, writing of the original draft; Fatih Bayansal: performed the ALD of ZnO on cotton samples; Mahmoud Aboelkheir: performed initial characterization of the ALD-coated samples; Lindsay Wolverton, Isabelle Reitz, and Nishka Avunoori: performed antibacterial activity and cell viability tests; Rimi Chowdhury: supervision of antibacterial and cell viability tests and writing the related part; Necmi Biyikli: supervision of ALD part; Tamer Uyar: conceptualization, methodology, supervision, project administration, funding, writing – review and editing.

## Conflicts of interest

The authors declare that they have no conflict of interest.

## Supplementary Material

NA-OLF-D6NA00078A-s001

## Data Availability

The data that support the findings of this study are available from the corresponding author upon reasonable request. Supplementary information (SI) is available. See DOI: https://doi.org/10.1039/d6na00078a.
