# Supplementary material for: Durable multifunctional cotton textiles through conformal ZnO nanocoatings by atomic layer deposition
Source: Nanoscale Adv. 2026 Sep 2;8(20):6038–55. doi: 10.1039/d6na00078a (PMC13592208; doi:10.1039/d6na00078a)
Supplement: Supplementary file 1 [file NA-008-D6NA00078A-s001.pdf]

Supplementary Information (SI) for Nanoscale Advances.
This journal is © The Royal Society of Chemistry 2026

Supplementary Information

Durable Multifunctional Cotton Textiles Through Conformal ZnO Nanocoatings by Atomic Layer Deposition

Md Sazid Bin Sadeque,[a] Fatih Bayansal,[b,c] Mahmoud Aboelkheir,[a] Lindsay Wolverton,[d] Isabelle Reitz,[d] Nishka Avunoori,[d] Rimi Chowdhury,[d] Necmi Biyikli,[b,c] and Tamer Uyar,[a]*

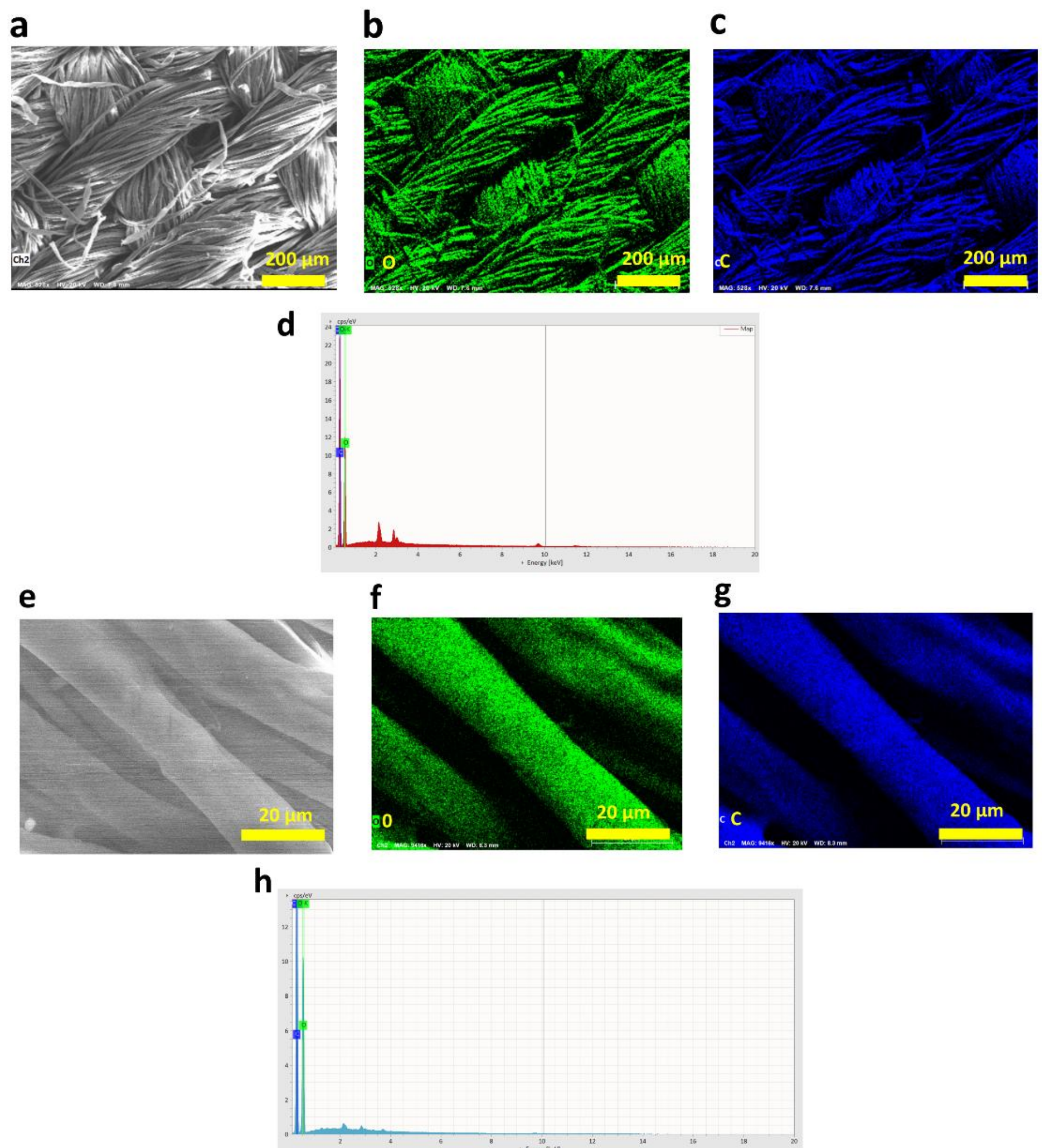

Figure. S1 a-c SEM and EDX elemental mapping of uncoated cotton fabric showing the characteristic rough, fibrillar morphology of cellulose fibers. d Corresponding EDX spectrum showing only C and O peaks, consistent with cellulose composition. e High-magnification SEM image of an individual uncoated cotton fiber. f,g Elemental

mapping of the single fiber showing homogeneous carbon and oxygen distribution. h EDX spectrum of the single fiber confirms the absence of Zn signals, verifying the uncoated state prior to ALD processing.

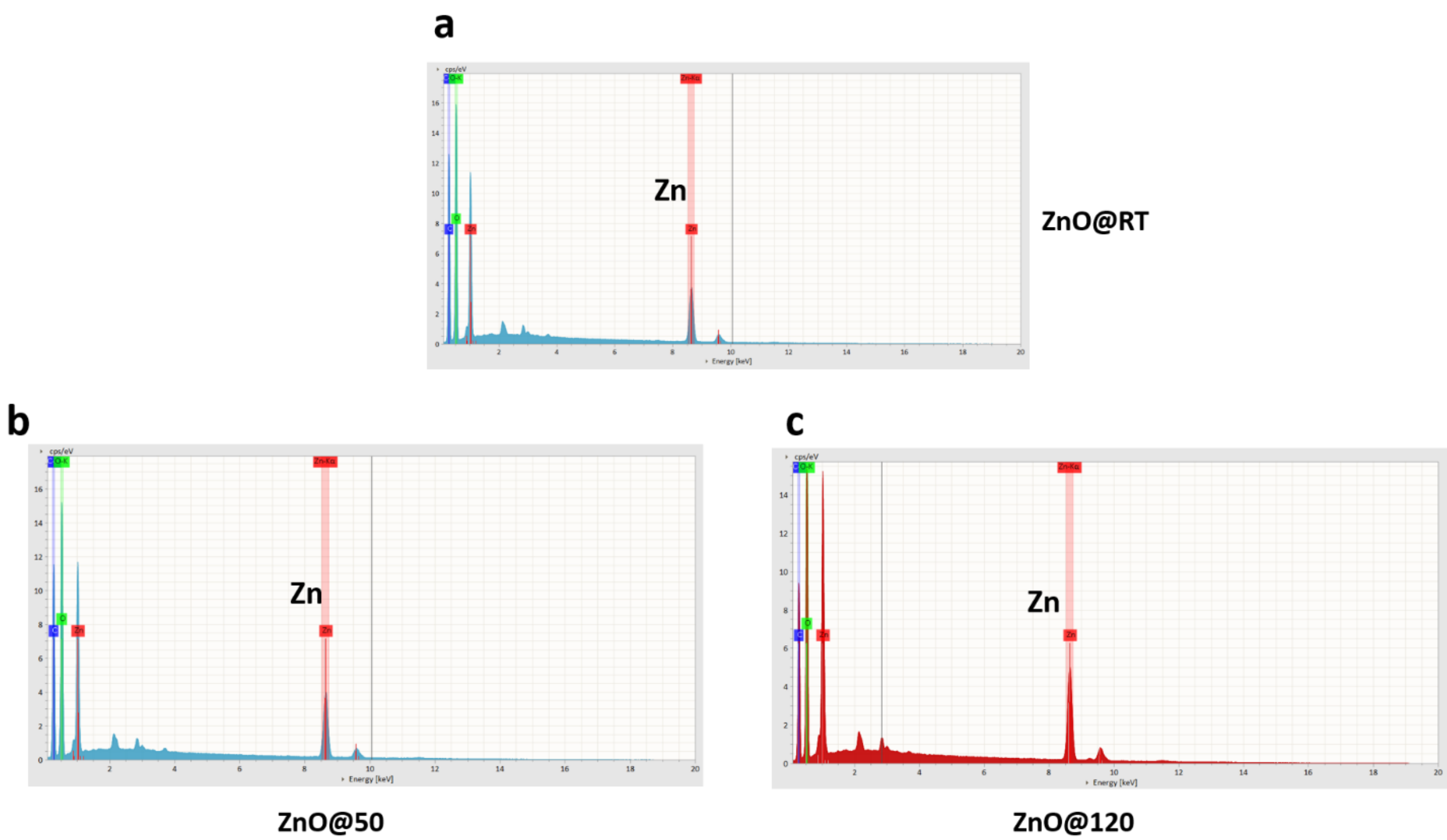

Figure. S2 EDX spectra of ZnO-coated cotton fabrics deposited at different ALD temperatures. a ZnO@RT showing weak Zn peaks, indicating limited nucleation and low-temperature film growth. b ZnO@50 demonstrating stronger Zn signals and improved coating formation compared to RT deposition. c ZnO@120 exhibiting the most intense Zn peaks, confirming enhanced ZnO crystallinity and uniformity at elevated temperature.

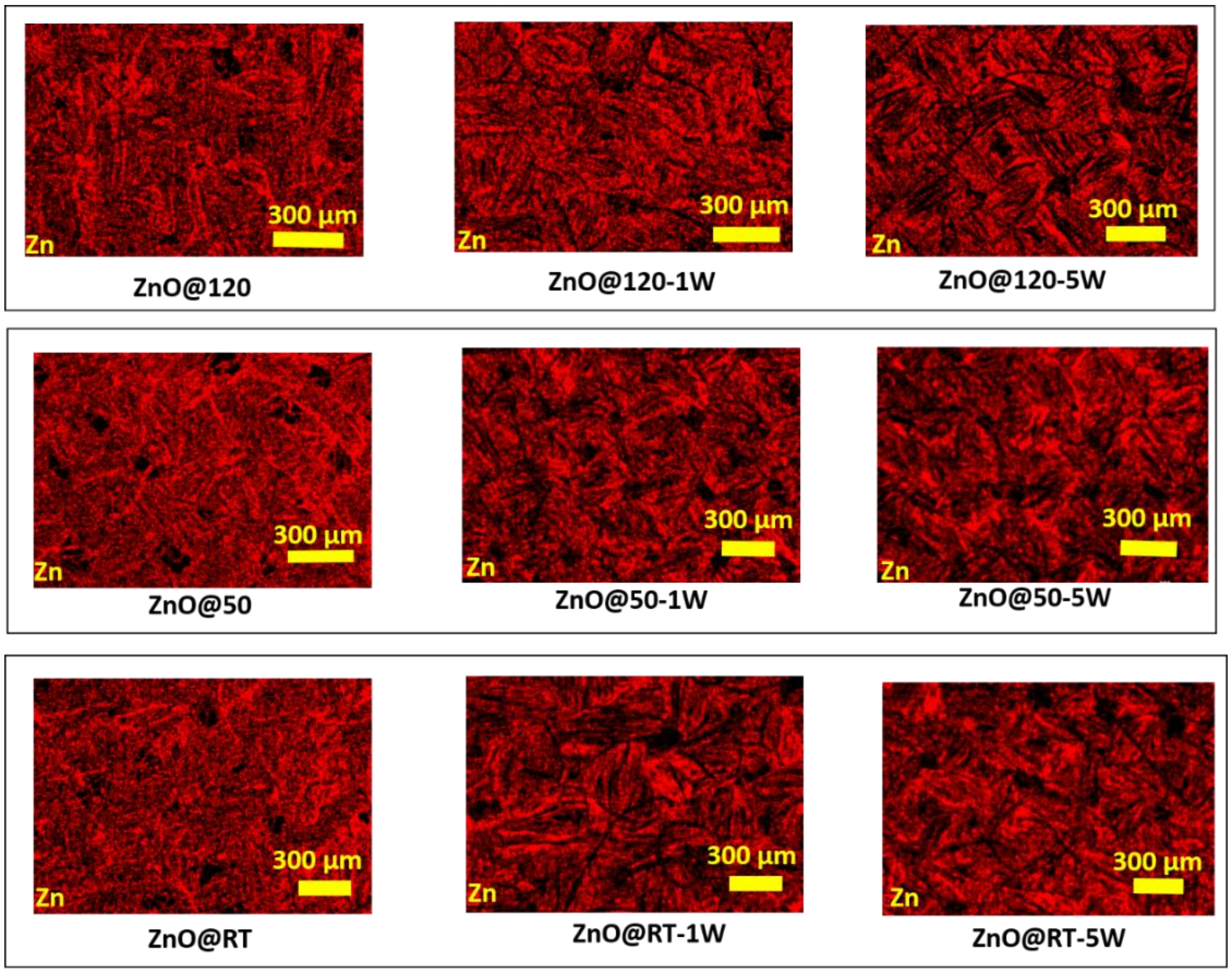

Figure. S3 Zn elemental mapping (EDX) of ZnO-coated cotton fabrics before and after washing.

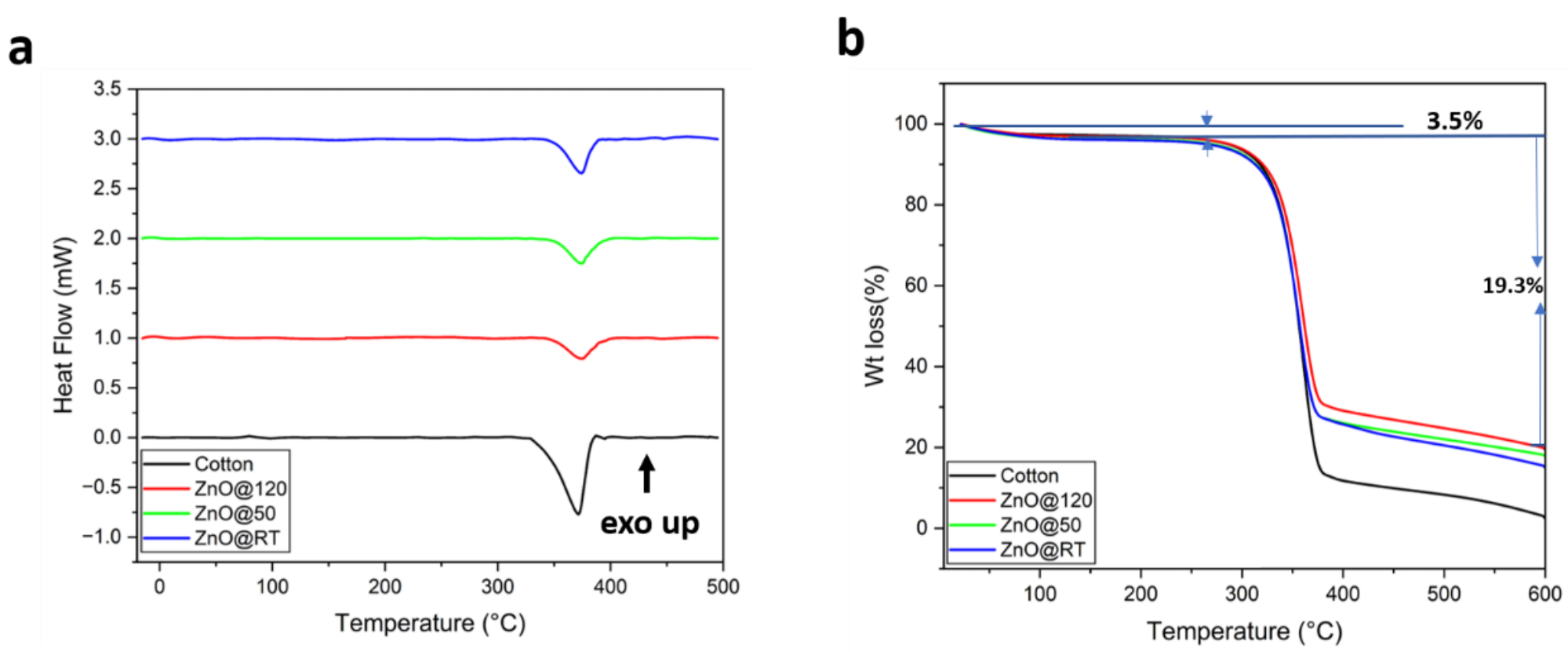

Figure. S4 Thermal analysis. a. DSC thermograms of uncoated and ZnO-coated cotton fabrics. b. Thermogravimetric analysis (TGA) of uncoated cotton and ZnO-coated cotton samples.

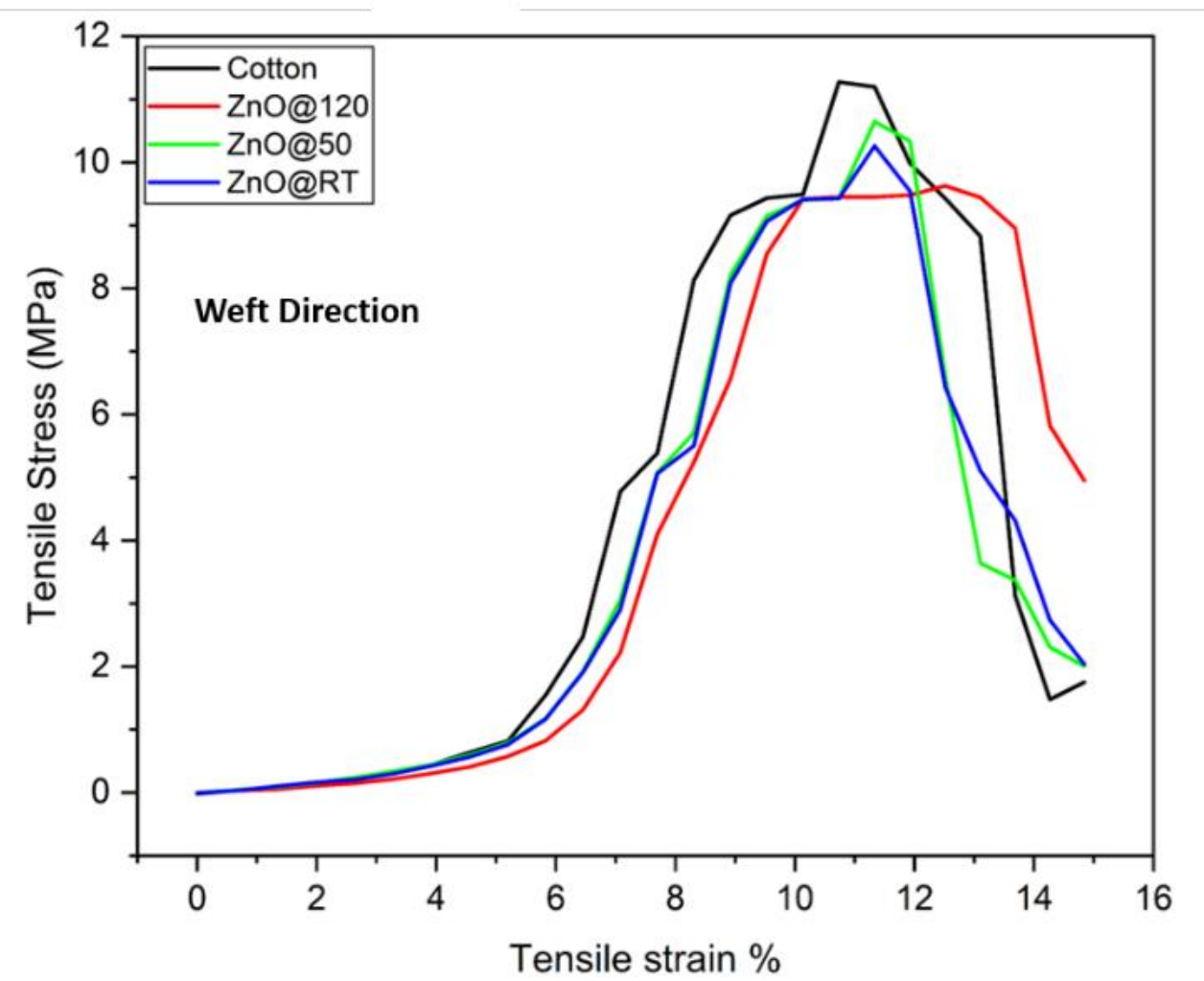

Figure. S5 Tensile stress–strain curves (weft direction) for uncoated and ZnO-coated cotton fabrics.

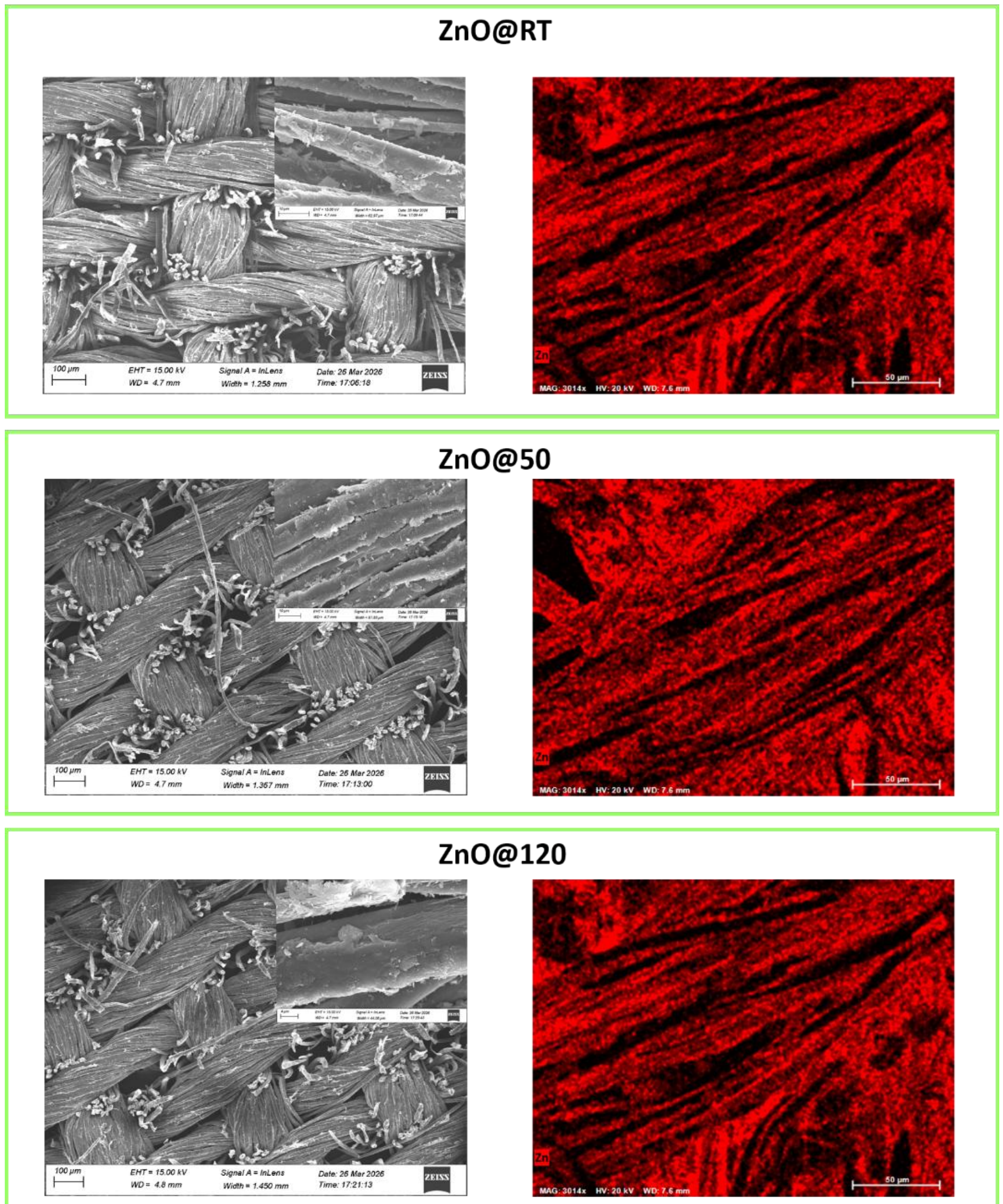

Figure. S6 SEM analysis on the coated samples after the Martindale Abrasion Test.

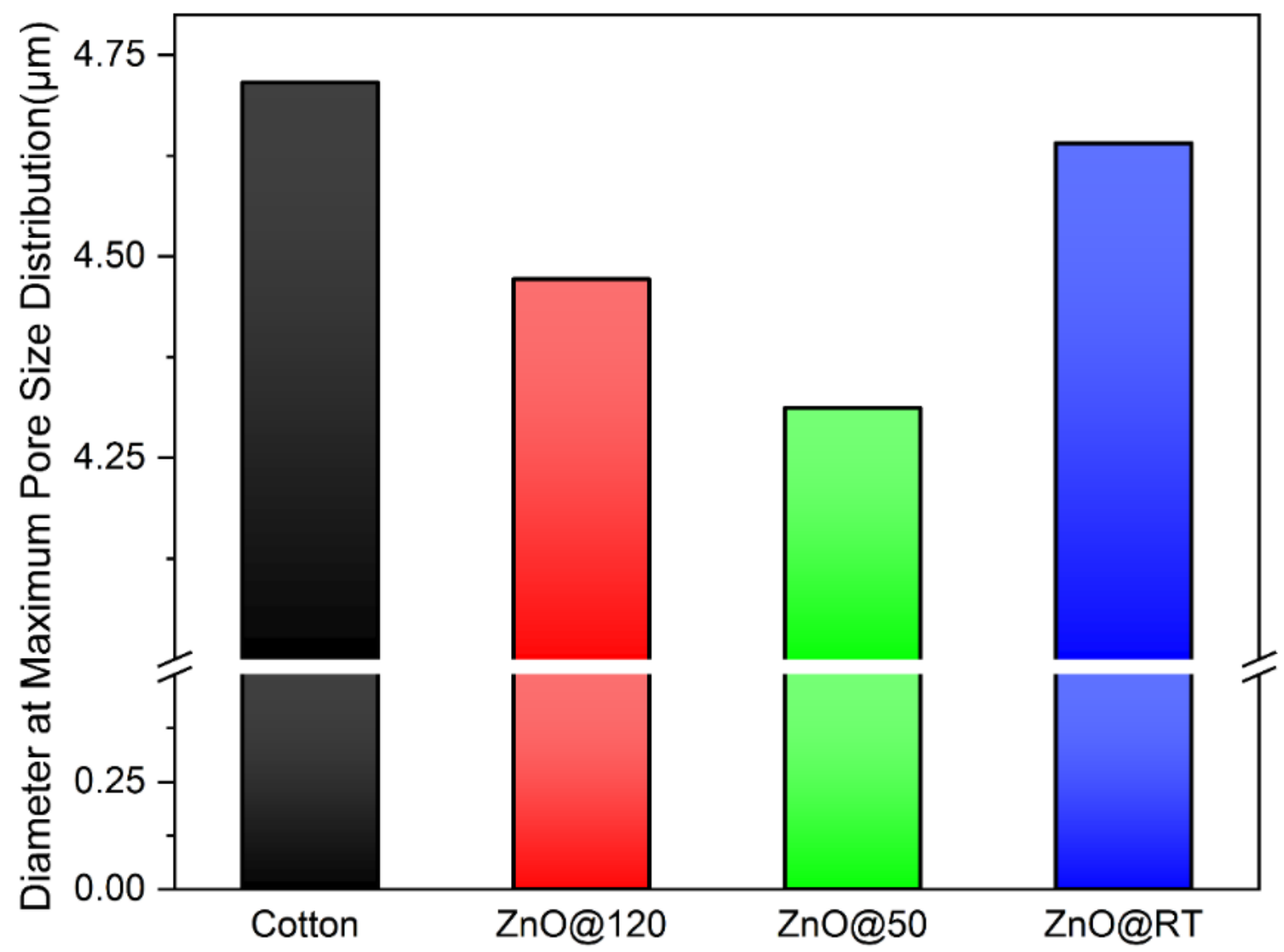

Figure. S7 Maximum pore diameter values extracted from porometry measurements.

a

Tolerances:	DL* tol	Da* tol	Db* tol	DC* tol	DH* tol	P/F tol	Margin	l:c
D65-10	2.58	0.66	0.70	0.70	0.66	1.00	0.10	2.00

Standard Name	L*	a*	b*	C*	h°
only cotton	95.41	-0.14	2.46	2.46	93.37

b

Trial Name	L*	a*	b*	C*	h°	DE*	DL*	Da*	Db*	DC*	DH*	DEcmc	P/F DEcmc
ZnO@RT	95.38	-0.45	2.78	2.81	99.13	0.44	-0.02 D	-0.30 G	0.32 Y	0.35 B	0.26 G	0.57	Passed
ZnO@50	95.37	-1.04	4.19	4.32	103.90	1.95	-0.03 D	-0.89 G	1.74 Y	1.86 B	0.60 G	2.48	Failed
ZnO@120	94.14	-1.66	8.31	8.48	101.28	6.18	-1.26 D	-1.51 G	5.86 Y	6.02 B	0.63 G	7.67	Failed

c

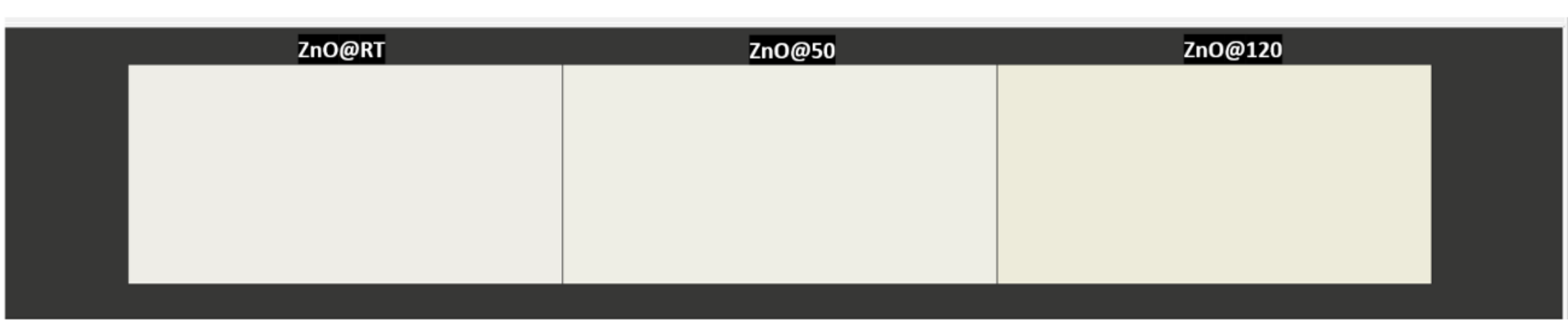

Figure. S8 Colorimetric analysis of uncoated cotton and ZnO@RT, ZnO@50, ZnO@120 samples. (a) L^*, a^*, b^* value of uncoated cotton fabric. (b) L^*, a^*, b^* value for the coated samples. (c) Visual representation of the shade difference of the coated samples at different temperatures.

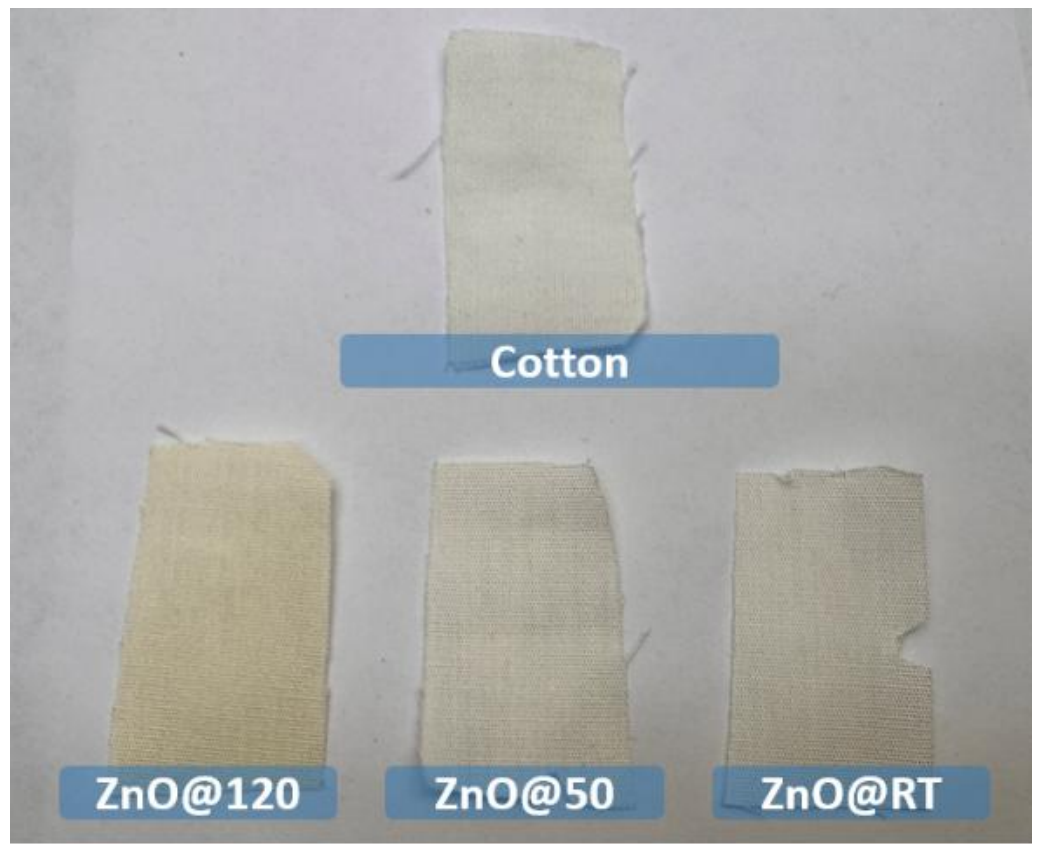

Figure. S9 Photographs of uncoated and ZnO-coated cotton fabrics unwashed state, showing slight visual yellowing for the cotton fabric at higher ALD temperatures.

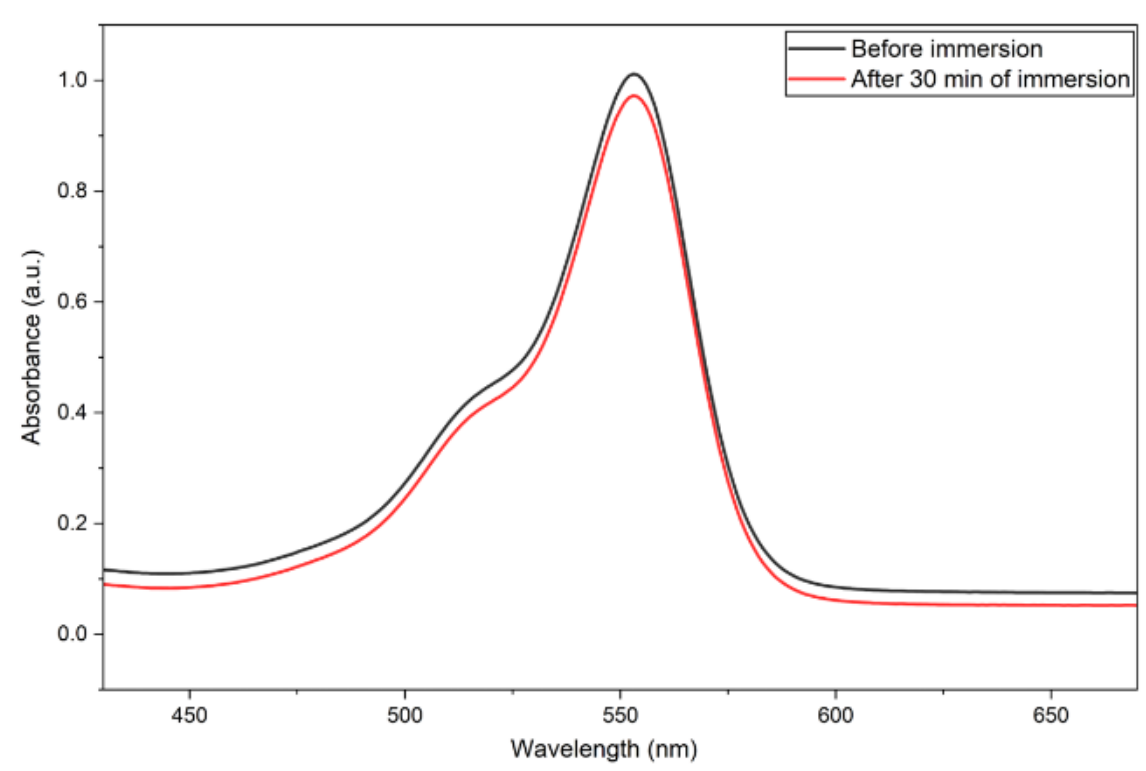

Figure. S10 Absorbance spectra of Rhodamine B solution before and after 30 min immersion of ZnO@120-5W, confirming negligible dye adsorption by the woven cotton substrate prior to photocatalytic testing.

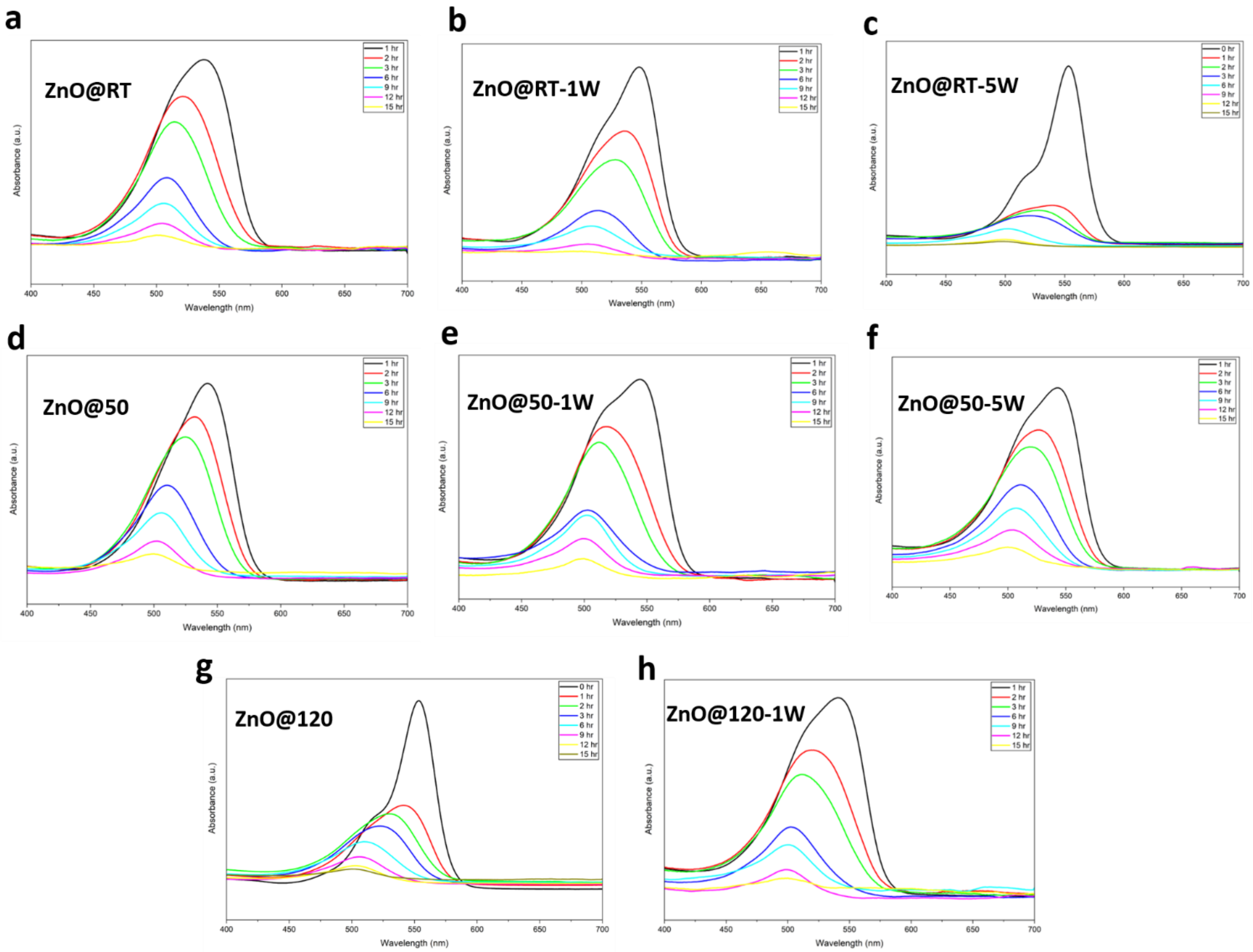

Figure. S11 UV–Vis absorbance spectra for ZnO@RT, ZnO@50 and ZnO@120 samples for 1 wash or 5 wash condition in RhB solution for 15 hr.

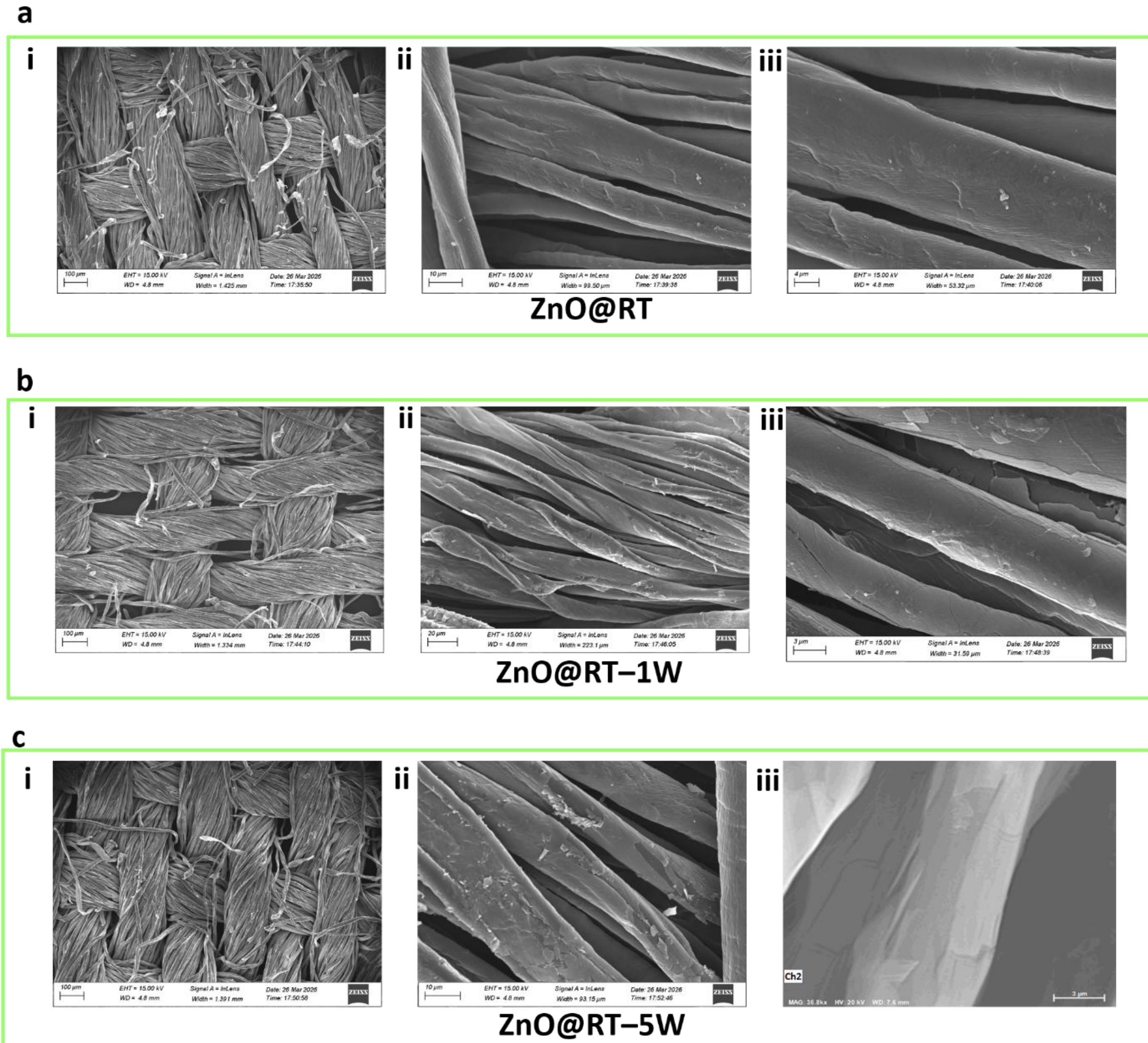

Figure. S12 The SEM analysis of (a) ZnO@RT, (b) ZnO@RT-1W, and (c) ZnO@RT-5W samples at different magnifications – (i) low magnification, (ii)high magnification and (iii) higher magnification.

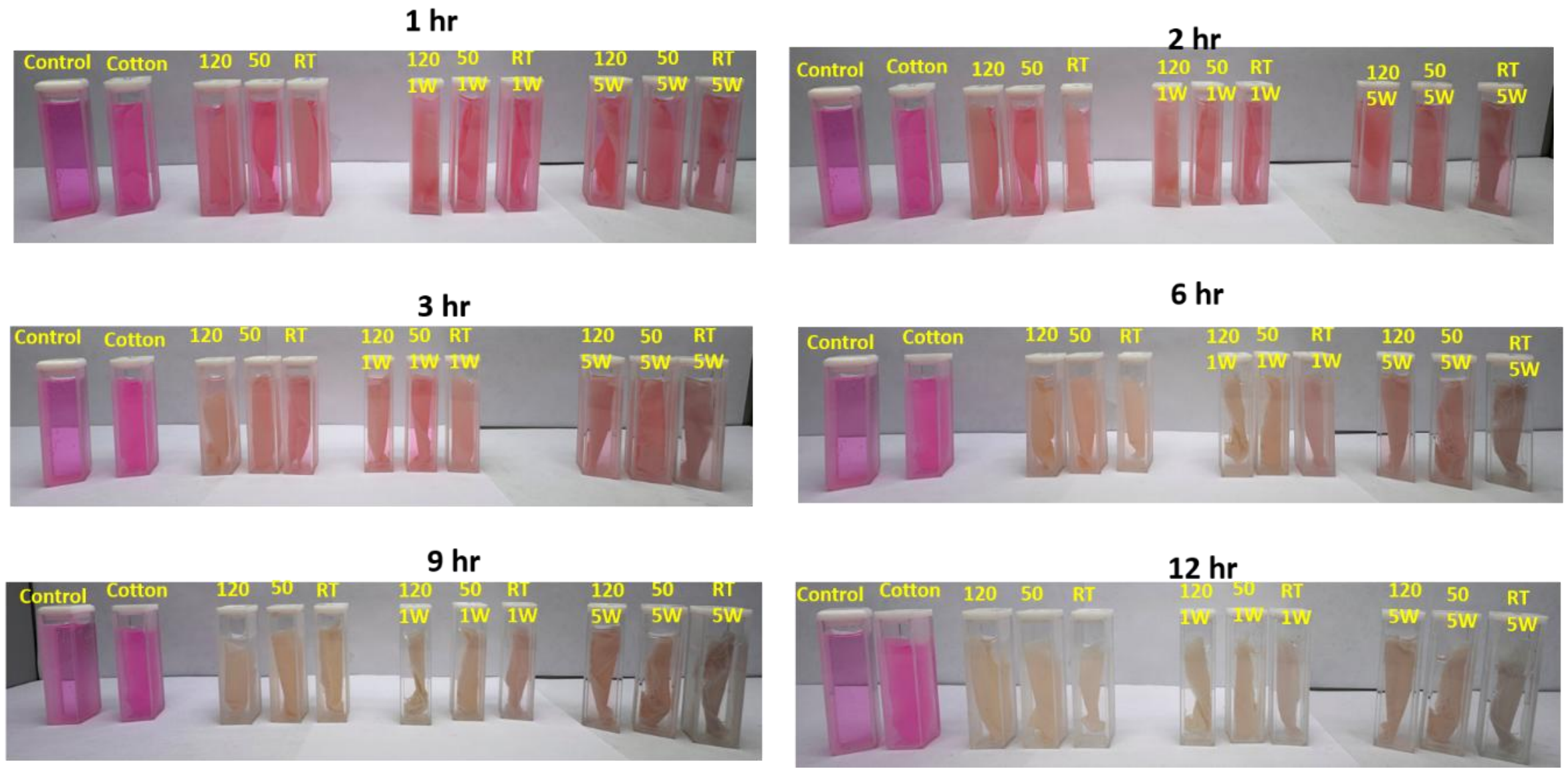

Figure. S13 Time-lapse visual comparison of Rhodamine B (RhB) degradation under 365 nm. Images captured at 1, 2, 3, 6, 9, and 12 hours show rapid fading of dye in all ZnO-coated samples.

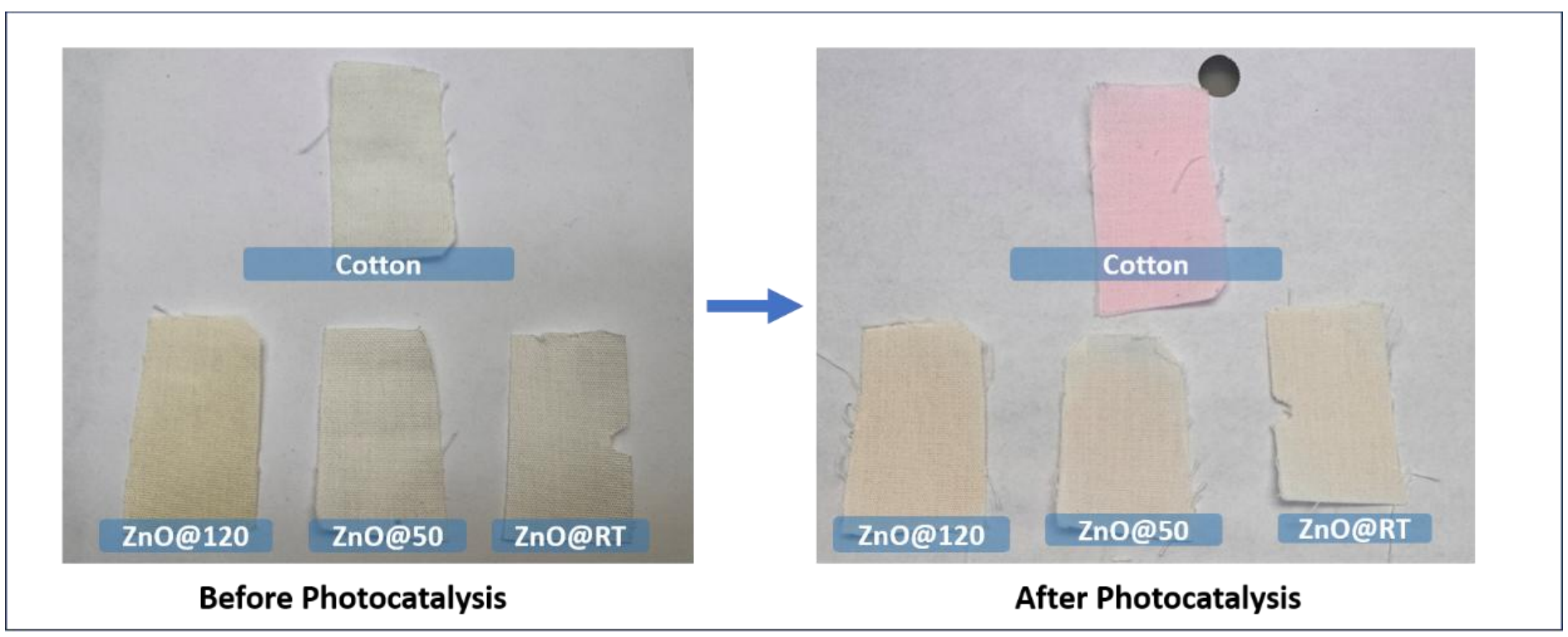

Figure. S14 Photographs of uncoated and ZnO-coated cotton fabrics unwashed samples before and after photocatalytic testing in RhB solution.

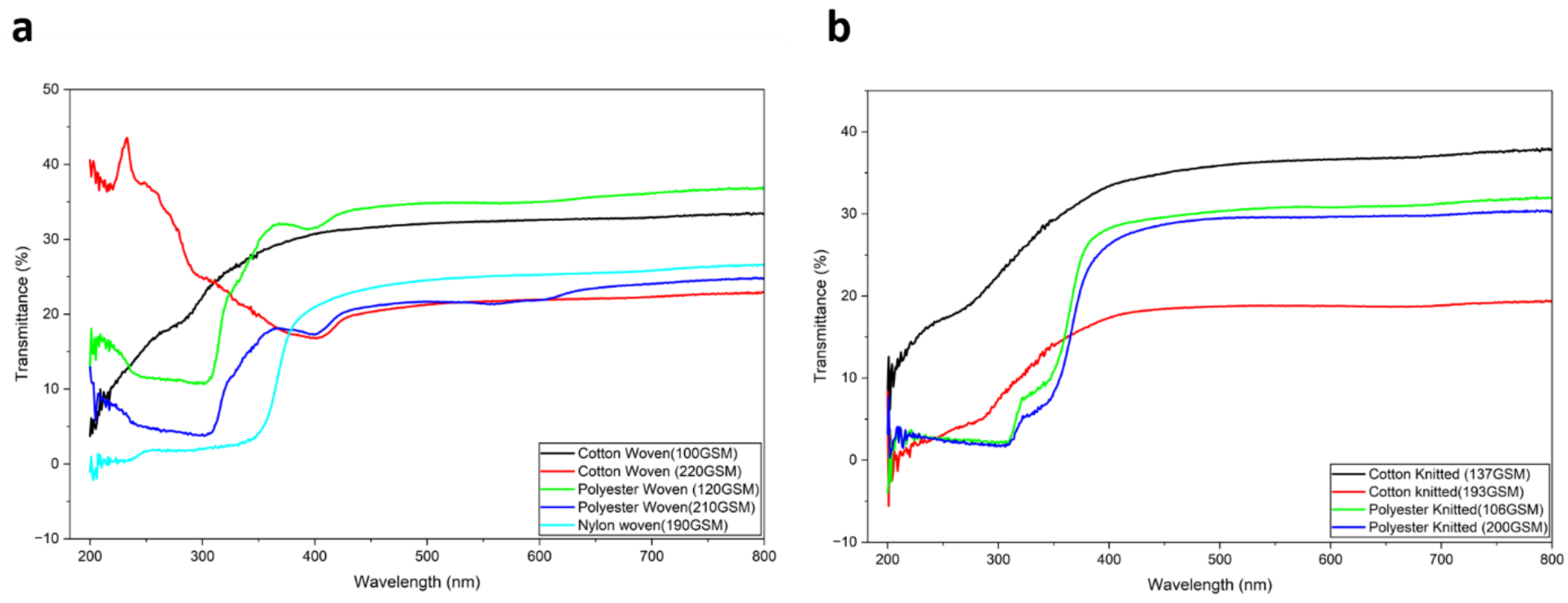

Figure. S15 UV blocking properties of various woven (a) and knitted (b) fabrics at different GSM.

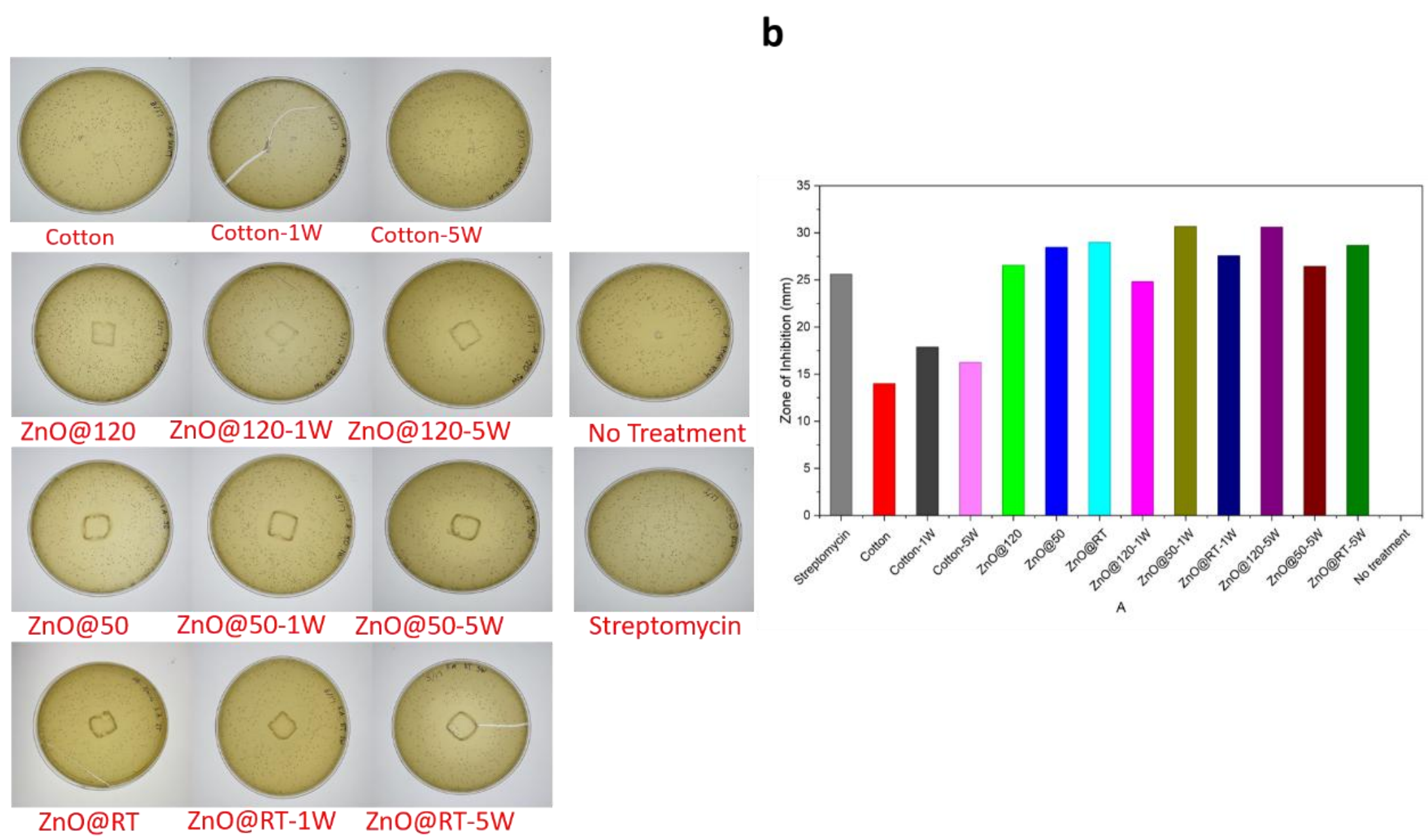

Figure. S16 (a) Disk diffusion assay of uncoated and ZnO-coated cotton fabrics against S. aureus. (b) Inhibition zone diameters showing that ZnO-coated fabrics produced larger inhibition zones than uncoated cotton.

Table S1 ZnO ALD on traditional textile

Fabric	Nano coating	Functionality	Year
Cotton (Woven)	ZnO	Photocatalysis, UV protection, Antibacterial activity, Thermal Comfort	This work
Polyester	Al_2O_3 and ZnO	Tactile sensor	2024[1]
Silk	ZnO	antibacterial	2023[2]

polypropylene (PP), Cotton	ZnO	Functional coating	2022[3]
Fiber glass	ZnO	Photocatalysis	2021[4]
polyamide 66	TiO_2 and ZnO	photocatalytic activity and antibacterial	2021[5]
cotton	ZnO	Thermoelectric	2020[6]
Cotton and viscose	ZnO and Al_2O_3	Antibacterial fabric	2019[7]
Cotton	ZnO	Antibacterial fabric	2019[8]
viscose	ZnO	UV protection	2018[9]
Cotton	ZnO	Thermoelectronics	2017[10]
nylon	ZnO	Conductivity and touch-sensor	2015[11]
Fiberglass	ZnO	Photocatalysis	2014[12]
nylon-6 and polypropylene	ZnO	Patterning	2014[13]
Cotton	ZnO	All-Fiber" Capacitors	2011[14]

Table S2: ALD study on traditional multi-functional textiles

Fabric	Nano coating	Functionality	Year	UPF	Antibacterial	Self-Cleaning
Cotton (Woven)	ZnO	Photocatalysis, UV protection, Antibacterial activity, Thermal Comfort	This work	UPF 37-40		Almost 80% dye(RhB) removal in 3 hr
Silk	TiO_2/ Al_2O_3	UV protection	2025[15]	160		
Meltblown nonwoven polypropylene	TiO2	Antibacterial and photocatalytic activity	2024[16]		4-fold increase in E. coli log inactivation	
Silk	ZnO	Antibacterial	2023[17]		Inhibition zone 15.1 mm against *E. coli*	
Fiber glass (woven)	TiO_2	Photocatalysis	2022[18]			Over 70% MB reduction in 4 hr
Cotton	Ag	Antibacterial	2022[19]		29 mm inhibition zone	
Cellulose	ZnO/Al_2O_3	Antibacterial	2022[20]		*E.coli* inactivation within 30 min UVA light irradiation	
Fiber glass (woven)	ZnO	Photocatalysis	2021[4]			Over 95% MB reduction in 4 hr
polyamide 66	TiO_2 and ZnO	photocatalytic activity and antibacterial	2021[5]			

Cotton and viscose	ZnO/ Al_2O_3	Antibacterial	2019[21]		Upto 78% inhibition rate against *E. coli*	
viscose	ZnO	UV protection	2018[9]	91% reduction of UV radiation		
Silk	TiO_2	UV protection	2015[22]	Approx. 70%UV absorption at 325nm		
polyamide/ aramid blend	TiO_2/ Al_2O_3	UV protection	2015[23]			

Table S3 Quantitative EDX analysis of ZnO-coated cotton fabrics before and after laundering cycles.

	Before Wash	After 1 Wash	After 5 Wash
Zn Mass Norm. %			
ZnO@120	32.81	23.67	18.61
ZnO@50	26.97	16.75	16.59
ZnO@RT	25.06	19.68	15.87
Zn Atom Norm. %			
ZnO@120	9.39	6.07	4.53
ZnO@50	7.23	4.67	4.31
ZnO@RT	6.5	5.68	4.05

Table S4 Antibacterial activity of ZnO-coated fabric samples by disk diffusion assay against E. coli and S. aureus

	Streptomycin	No treatment	Cotton	Cotton-1W	Cotton-5W	ZnO @120	ZnO @120-1W	ZnO @120-5W	ZnO @50	ZnO @50-1W	ZnO @50-5W	ZnO @RT	ZnO @RT-1W	ZnO @RT-5W
E. coli	32.455	0	14.512	16.317	15.539	25.183	22.142	25.091	24.76	24.356	27.617	25.096	25.055	24.76
S. aureus	25.605	0	14	17.858	16.243	26.557	24.805	30.617	28.458	30.665	26.427	28.986	27.566	28.674

References

1 M.-Y. Liu, Y.-F. Zhang, L.-X. Ou, L.-Y. Zhu, X.-Y. Wu, Y. Wang, Y. Gu, Y.-C. Chen and H.-L. Lu, *Appl. Mater. Today*, 2024, **37**, 102099.

2 X. Xiao, B. Zhang, P. Lyu, F. Chen, Q. Zhang, J. Sun, Z. Jiang, X. Liu and W. Xu, *Appl. Surf. Sci.*, 2023, **610**, 155487.

3 G. Gurbandurdyyev, K. Mistry, L.-V. Delumeau, J. Y. Loke, C. H. Teoh, J. Cheon, F. Ye, K. C. Tam and K. P. Musselman, *ChemNanoMat*, 2023, **9**, e202200498.
4 S. Islam and H. I. Akyildiz, *J. Mater. Sci. Mater. Electron.*, 2021, **32**, 27027–27043.
5 H. I. Akyildiz, S. Diler and S. Islam, *J. Vac. Sci. Technol. A*, 2021, **39**, 022405.
6 G. Marin, R. Funahashi and M. Karppinen, *Adv. Eng. Mater.*, 2020, **22**, 2000535.
7 M. C. Popescu, C. Ungureanu, E. Buse, F. Nastase, V. Tucureanu, M. Suchea, S. Draga and M. A. Popescu, *Appl. Surf. Sci.*, 2019, **481**, 1287–1298.
8 R. U. Puvvada, J. P. Wooding, M. C. Bellavia, E. K. McGuinness, T. A. Sulchek and M. D. Losego, *JOM*, 2019, **71**, 178–184.
9 M. C. Popescu, F. Nastase, I. Mihalache, M. A. Banu, V. Tucureanu, C. Ungureanu, B. C. Tincu and R. M. Tomescu, in *Advanced Topics in Optoelectronics, Microelectronics, and Nanotechnologies IX*, SPIE, 2018, vol. 10977, pp. 62–69.
10 A. J. Karttunen, L. Sarnes, R. Townsend, J. Mikkonen and M. Karppinen, *Adv. Electron. Mater.*, 2017, **3**, 1600459.
11 W. J. Sweet III, C. J. Oldham and G. N. Parsons, *J. Vac. Sci. Technol. A*, 2014, **33**, 01A117.
12 H. I. Akyildiz, E. Yigit, A. B. Arat and S. Islam, *J. Photochem. Photobiol. Chem.*, 2024, **448**, 115308.
13 W. J. I. Sweet, C. J. Oldham and G. N. Parsons, *ACS Appl. Mater. Interfaces*, 2014, **6**, 9280–9289.
14 Jesse. S. Jur, W. J. Sweet III, C. J. Oldham and G. N. Parsons, *Adv. Funct. Mater.*, 2011, **21**, 1993–2002.
15 T. Xing, A. He, Z. Du, Y. Zhang, Y. Luo, Z. Huang, M. Wang, Z. Shi, A. Tong, S. Qiao, X. Liao, J. Bai, H. Pan, Z. Liang, W. Ke, H. Yu and F. Chen, *ACS Appl. Mater. Interfaces*, 2025, **17**, 25961–25975.
16 A. G. Aragon, J. A. Cárdenas Sánchez, C. Zimeri, E. Shim, X. Fang and K. R. L. Young, *Environments*, 2024, **11**, 92.
17 X. Xiao, B. Zhang, P. Lyu, F. Chen, Q. Zhang, J. Sun, Z. Jiang, X. Liu and W. Xu, *Appl. Surf. Sci.*, 2023, **610**, 155487.
18 S. Islam and H. I. Akyildiz, *J. Mater. Sci. Mater. Electron.*, 2022, **33**, 18002–18013.
19 H. I. Akyildiz, B. A. Yilmaz and S. Diler, *Fibers Polym.*, 2022, **23**, 2769–2779.
20 S. Mežnarić, I. Jelovica Badovinac, I. Šarić, R. Peter, M. Kolympadi Markovic, G. Ambrožić and I. Gobin, *J. Environ. Chem. Eng.*, 2022, **10**, 108095.
21 M. C. Popescu, C. Ungureanu, E. Buse, F. Nastase, V. Tucureanu, M. Suchea, S. Draga and M. A. Popescu, *Appl. Surf. Sci.*, 2019, **481**, 1287–1298.
22 X. Xiao, X. Liu, F. Chen, D. Fang, C. Zhang, L. Xia and W. Xu, *ACS Appl. Mater. Interfaces*, 2015, **7**, 21326–21333.
23 X. Xiao, X. Liu, G. Cao, C. Zhang, L. Xia, W. Xu and S. Xiao, *Polym. Eng. Sci.*, 2015, **55**, 1296–1302.
